# The last man standing is the most resistant: eliminating artemisinin-resistant malaria in Cambodia

**DOI:** 10.1186/1475-2875-8-31

**Published:** 2009-02-20

**Authors:** Richard J Maude, Wirichada Pontavornpinyo, Sompob Saralamba, Ricardo Aguas, Shunmay Yeung, Arjen M Dondorp, Nicholas PJ Day, Nicholas J White, Lisa J White

**Affiliations:** 1 Mahidol-Oxford Tropical Medicine Research Unit, Faculty of Tropical Medicine, Mahidol University, 3/F, 60th Anniversary Chalermprakiat Building, 420/6 Rajvithi Road, Rajthevee, Bangkok 10400, Thailand; 2 Centre for Clinical Vaccinology and Tropical Medicine, Nuffield Department of Clinical Medicine, Unversity of Oxford, Oxford, UK; 3 Instituto Gulbenkian de Ciência, Oeiras, Portugal; 4 Health Policy Unit, London School of Hygiene and Tropical Medicine, Keppel Street, London, UK

## Abstract

**Background:**

Artemisinin combination therapy (ACT) is now the recommended first-line treatment for falciparum malaria throughout the world. Initiatives to eliminate malaria are critically dependent on its efficacy. There is recent worrying evidence that artemisinin resistance has arisen on the Thai-Cambodian border. Urgent containment interventions are planned and about to be executed. Mathematical modeling approaches to intervention design are now integrated into the field of malaria epidemiology and control. The use of such an approach to investigate the likely effectiveness of different containment measures with the ultimate aim of eliminating artemisinin-resistant malaria is described.

**Methods:**

A population dynamic mathematical modeling framework was developed to explore the relative effectiveness of a variety of containment interventions in eliminating artemisinin-resistant malaria in western Cambodia.

**Results:**

The most effective intervention to eliminate artemisinin-resistant malaria was a switch of treatment from artemisinin monotherapy to ACT (mean time to elimination 3.42 years (95% CI 3.32–3.60 years). However, with this approach it is predicted that elimination of artemisinin-resistant malaria using ACT can be achieved only by elimination of all malaria. This is because the various forms of ACT are more effective against infections with artemisinin-sensitive parasites, leaving the more resistant infections as an increasing proportion of the dwindling parasite population.

**Conclusion:**

Containment of artemisinin-resistant malaria can be achieved by elimination of malaria from western Cambodia using ACT. The "last man standing" is the most resistant and thus this strategy must be sustained until elimination is truly achieved.

## Background

The Thai-Cambodian border area is historically the source of the global diaspora of anti-malarial drug resistance. Resistance to chloroquine and sulphadoxine-pyrimethamine in *Plasmodium falciparum *originated there, spread across Asia and Africa, and caused millions of deaths [1]. The increase in malaria mortality is now being reversed where effective vector control measures and anti-malarials, principally artemisinin-based combination therapy (ACT), are being deployed [2]. Current initiatives to eliminate malaria are critically dependent on their continued efficacy.

In the 2006, WHO Guidelines for the Treatment of Malaria [3] ACT became the recommended first-line treatment for uncomplicated *Plasmodium falciparum *malaria in all endemic areas. Intravenous artesunate became the treatment of choice for severe malaria, except for children in Africa (where studies are in progress) [3,4]. These recommendations for the large-scale use of artemisinin derivatives were based on their excellent tolerability, safety, and reliable efficacy. Recent data from western Cambodia provides the first objective evidence that efficacy of this essential drug class may be declining [5-7]. Cure rates with ACT have been worse in this area than anywhere else. This was attributed initially to resistance to the partner drugs (mefloquine [8] and lumefantrine [9]), but recent detailed studies show that parasite clearance times following standard doses of artesunate in uncomplicated falciparum malaria are significantly longer than elsewhere in the world [5-7]. Artemisinins have been available as monotherapies in western Cambodia for over 30 years in a variety of forms and doses, whereas in most countries, other than China where they were discovered, they have been a relatively recent introduction [5]. The extended period of often sub-optimal use and the genetic background of parasites from this region have created a dangerous milieu [10]. If reduced *in-vivo *parasitological efficacy is the first sign of artemisinin resistance then immediate action should be taken to prevent the spread of these parasites elsewhere [11]. Loss of these drugs would be a disaster for global malaria control and elimination prospects as there are no obvious replacements emerging from the development pipeline in the near future [12]. Fortunately malaria in western Cambodia can be considered as affecting a land island with no major contiguous connections to other malaria endemic areas [13]. The private sector is the main source of anti-malarial drugs in Cambodia. Self-treatment with short (inadequate) courses of oral artemisinin monotherapies has been common [14]. Many other anti-malarials are available, including artesunate-mefloquine (MAS3), the nationally recommended first-line treatment for falciparum malaria since 2000. Several different interventions to contain and ideally eliminate the threat of artemisinin resistance are under consideration by the national malaria control programmes, the World Health Organization and malaria community [6].

The current debate centres around a number of questions including:

• Which anti-malarial should be used in different situations? Under consideration are atovaquone plus proguanil (AP) and/or dihydroartemisinin and piperaquine, both given for three days, plus one "gametocytocidal" dose of primaquine (APP).

• Who should receive these treatments? a) symptomatic patients only b) anyone with a positive blood film for malaria ('Mass Screening and Treatment', MSAT) c) the general population regardless of symptoms or blood film positivity ('Mass Drug Administration', MDA).

• How long will an intervention need to be continued? Longer interventions are more costly and difficult to sustain. The cost-effectiveness of each proposed intervention and the consequences of premature cessation have not yet been quantified.

• What is the added value of insecticide-treated bed nets (ITNs)?

**Figure 1:**
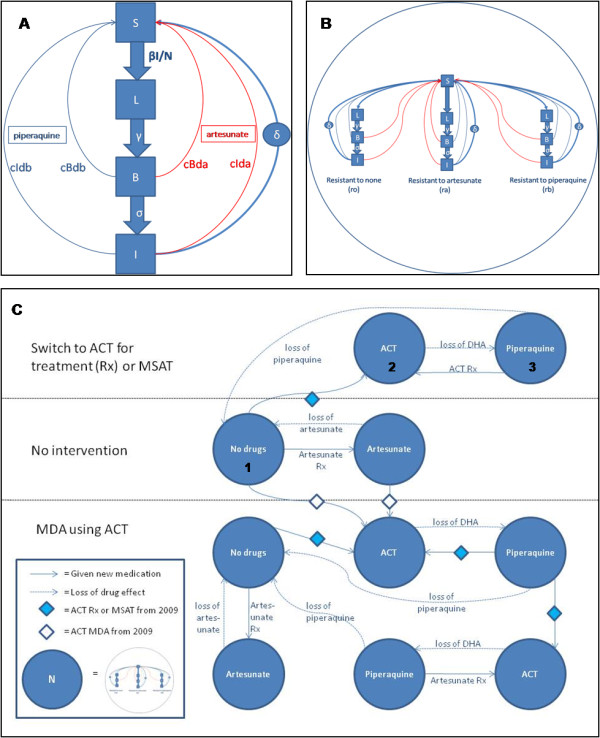
**Schematic diagram of the structure of the mathematical modeling framework**. The structure of the model is built up as follows: natural history and pharmacodynamics incorporated as a repeating unit (**A**) with four compartments – **susceptible **people, people with **liver stage **infection, people with noninfectious **blood stage **infection and people with **infectious **(blood) **stage **infection, rates of flow between these compartments (**βI/N**, **δ, γ **and **σ**) and rates of recovery due to each of artesunate and piperaquine treatment (**c_Bda_**, **c_Ida_**, **c_Bdb _**and **c_Idb_**) (Additional file 1- Table S2) is shown. The times to recovery (1/rate) following treatment are then adjusted by a multiplying factor (e_rada _or e_rbdb_) (0 ≤ e ≤ 1) depending on the degree of resistance to each drug, giving three possible linked variants of unit **A **(resistant to no drug, artesunate only and piperaquine only) making up a repeating pattern (**B**). Finally the population dynamics of transmission is shown in (**C**). This consists of multiple repetitions of (**B**) with different rates of flow between them at different time points depending on which treatments and interventions are used. For example, for individuals with blood stage infections to begin treatment with ACT, they will move from the 'No drugs' box (**1**) to the equivalent parts of an 'ACT' box (**2**) at a rate determined by the time to begin treatment. The dynamics in the 'ACT' box are different from the 'No drugs' box as these individuals will be subjected to faster rates of recovery due to the ACT. Each box is also subject to pharmacokinetic dynamics independent of infection dynamics. This is in the form of waning pharmacodynamic drug effect over time ('loss of...') with sequential loss of DHA and then piperaquine. This results in a percentage of the entire unit moving to a new box 'Piperaquine' (**3**) which again has different dynamics representing the effect of piperaquine on recovery rates. Interventions shown here are elimation of artemisinin monotherapy and replacement with ACT ('Switch to ACT') and MSAT and MDA with ACT. Each circle represents a population exposed to a particular drug or combination. Key: ACT = dihydroartemisinin/piperaquine combination therapy, Rx = treatment, DHA = dihydroartemisinin. (For more details, please see the Full Model Code in Additional File 2.)

These choices must be made now on the basis of available evidence to reduce the risk of resistance spreading westward. Containment beyond the western border of Thailand is probably impossible. There are insufficient data and insufficient time to undertake adequately powered clinical studies to inform these urgently needed decisions, so the relevant agencies are currently relying upon expert opinion. A mathematical modeling framework (see Figure 1 and Additional Files 1 and 2) of malaria in Cambodia focusing on the population dynamics of artemisinin resistance and its control was, therefore, developed to aid decision making [15].

## Methods

**Figure 2:**
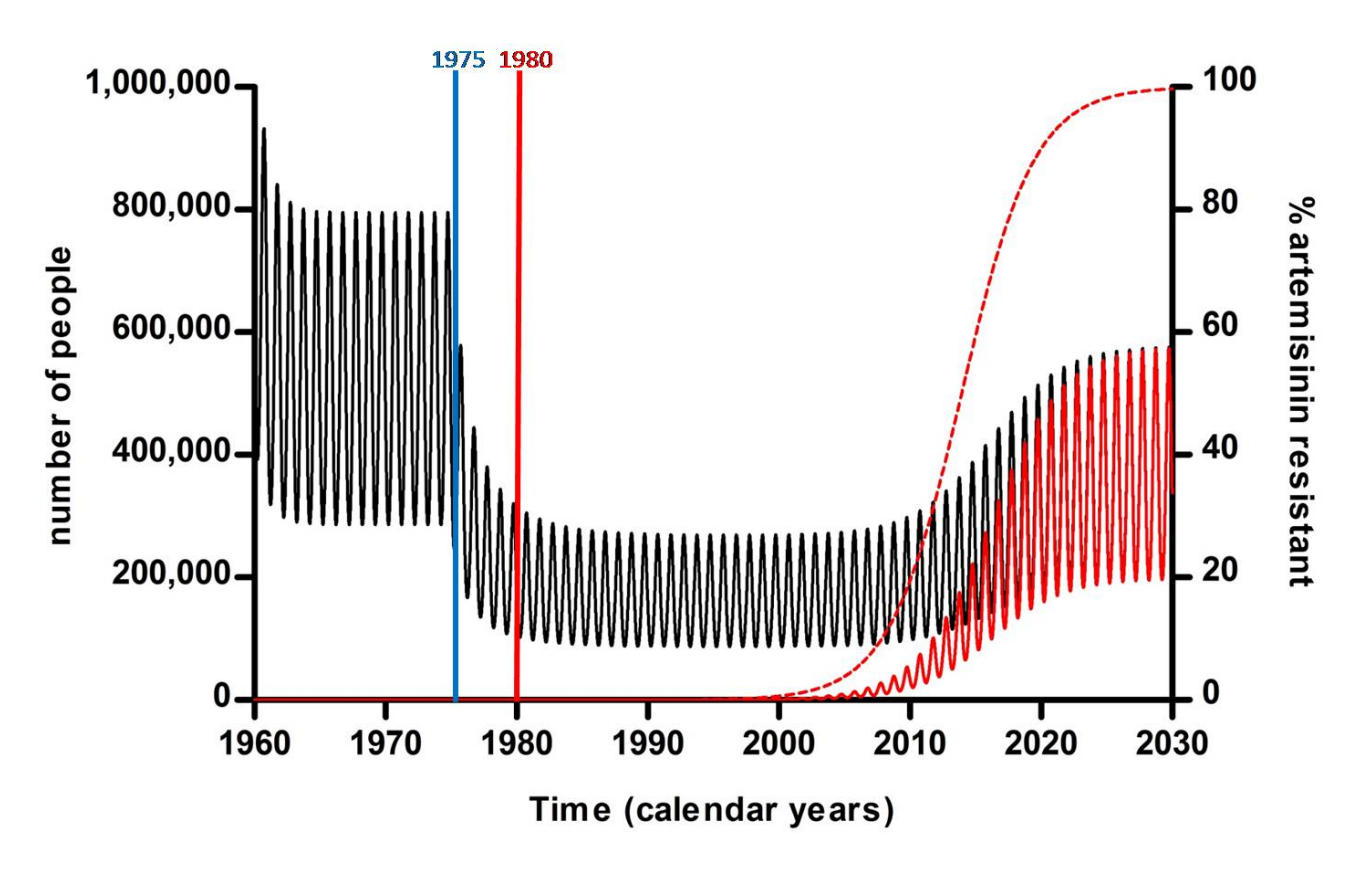
**Effect of continuing availability and use of artemisinin monotherapy on the total number of malaria infections (black line), the number of artemisinin-resistant infections (red line) and percentage of infections resistant to artemisinin (red dotted line) over time**. Artesunate monotherapy is introduced as treatment in 1975 and a single artemisinin-resistant infection in 1980.

The structure of the basic population dynamic model is shown in Figure 1. It is based on the malaria parasite life-cycle in humans and the responses of the various stages to drug treatment. A deterministic structure was used to conduct sensitivity analyses and to make an initial assessment of the relative effectiveness of the various containment interventions proposed. Results of interest were then confirmed in a stochastic framework with the same structure and parameters. Seasonality was incorporated to reflect the highly seasonal transmission intensity in this region. In order to maintain simplicity and therefore flexibility and interpretability of the model, a number of assumptions were made (Additional file 1-Table S1). The model was run from 1960–1975 to achieve a treatment free equilibrium for a population prevalence of malaria parasitaemia (of any density) of 16%. This equilibrium was calculated by the numerical solution of the treatment-free sub-model equilibrium equations. Artesunate monotherapy was then introduced as the only treatment in 1975 (when artesunate was first used in Cambodia). A single patient with artemisinin-resistant infection was introduced in 1980. By 2008, the mean population prevalence of malaria in the high transmission season was 7.5% (compared to estimate from field data of 7.4% [13] and the prevalence of artemisinin resistance in the model was 10.6% (expert opinion estimates this to be around 10%) (this whole process is shown in Figure 2). The interventions were then introduced in 2009, to reflect the current plans for containment.

The full list of parameters is shown in Table S2 (Additional File 1). The interventions that were introduced into the model, alone and in combination, were:

a) Eliminating the use of artemisinin monotherapies (artesunate in the model) and replacing them with ACT in the private sector (i.e. for symptomatic patients only).

b) One or more annual three-month pulses of MSAT with ACT or AP during the low transmission season.

c) One or more annual three-month pulses of MDA with ACT or AP during the low transmission season.

d) As for b) and c) plus primaquine.

e) Distribution of ITNs (or comparable treated materials).

For each pulse of MDA and MSAT, each patient received a three-day course of treatment (ACT or AP) on one occasion only.

## Results

### Continuing availability of artesunate monotherapy

The model predicts that if there is no intervention, and use of artemisinin monotherapies continues, there will be an exponential rise in the proportion of resistant infections and a slowly increasing prevalence of infection. By 2030, the model predicts that the prevalence of malaria will have doubled compared to 2008 and resistance to the artemisinins will be approaching 100% (Figure 2).

### Eliminating artemisinin resistance

The model predicts that it is possible to achieve elimination of artemisinin resistance using medications alone, but in all scenarios elimination of malaria is required in order to eradicate artemisinin resistance.

**Figure 3:**
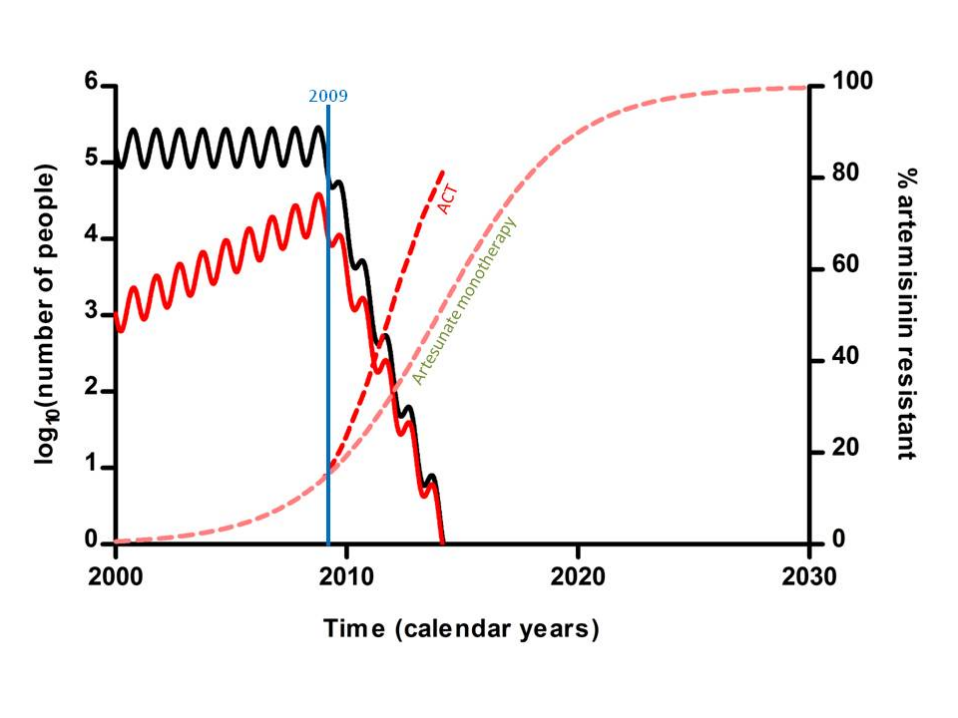
**Effect of eliminating artesunate monotherapy and replacement with ACT in 2009 for treatment of symptomatic cases on the total number of malaria infections (black line), the number of artemisinin-resistant infections (red line) and the percentage of infections resistant to artesunate (dotted lines, pink = artesunate red = ACT) (mean-field approximation)**.

**Figure 4:**
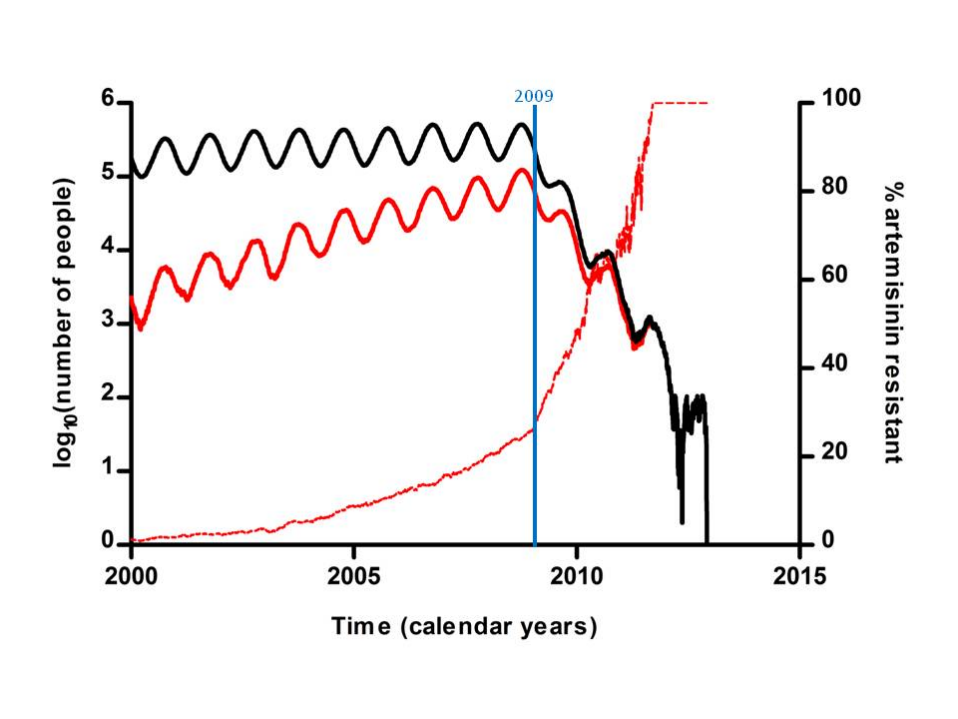
**Example of a single stochastic output illustrating the effect of a switch of treatment to ACT in 2009**.

The most effective single intervention to achieve elimination of artesunate resistance is the elimination of inadequate courses of artesunate monotherapy and replacement with ACT with high coverage (a). This would be achieved if all monotherapies were actively replaced by ACT in the private sector and there was adequate continued supply of good quality, affordable ACT. Using this strategy and with an introduction of the ACT over three months it is possible in the model to achieve elimination of artemisinin-resistant malaria within four years in 70% of cases (mean time to elimination 3.42 years (95% CI 3.32–3.60 years), (Figure 3). The stochastic model was run 200 times and elimination was achieved with this strategy in 100% of cases, suggesting this strategy is highly likely to be successful. The downside is that, because a lot of artemisinin is being used, the prevalence of artemisinin resistance just before elimination drastically increases. It reaches 82% in the deterministic model and 100% in 58% of cases in the stochastic model (Figure 4 illustrates one of these cases).

The proportion of infections resistant to artemisinins then remains at this high level provided that there is no importation of new infections from elsewhere. This suggests that if an intervention were to fail, or was discontinued prematurely, the prevalence of resistance would be much higher subsequently than if no intervention had taken place.

If artemisinin monotherapies remain available, one three-month pulse of MSAT or MDA with either ACT or AP (b or c) reaching 80% of the infected population would have a significant short-term impact, but a negligible long-term impact on the numbers of resistant infections. This is because although such interventions reduce infections they are not sufficient to completely interrupt transmission at the population level. For example, AP MSAT is the most effective of these strategies but one pulse of this only produces a 35% reduction in total malaria infections and 31% reduction in the number of artemisinin-resistant infections. If a three-month pulse of MSAT or MDA is used in addition to a switch to ACT for treatment then there is negligible additional effect. Repeated annual three-month pulses of MSAT or MDA are also insufficient to achieve elimination when used alone, regardless of the drugs used. This is because the number of infections increases again in the other nine months of the year with an increasing proportion of artemisinin-resistant infections each time due to ongoing artemisinin use. When used in this way, MSAT is more effective than MDA and AP has greater impact than ACT. For example, the maximum effect of long-term annual MSAT with AP on the numbers of artesunate resistant infections is at two years. At this time, the decrease in malaria infections is 55% and artemisinin-resistant infections have fallen to 39% compared to 2009. Following this trough, the number of artemisinin-resistant infections rises again although the total number of malaria infections does not reach a minimum until after 7 years (80% below that for 2009). For comparison, if ACT MSAT is used, the lowest number of artesunate resistant infections is in the first year with a 32% decrease in malaria prevalence and 31% fall in artesunate resistant infections compared to 2009. The number of artesunate resistant infections then increases again but the total number of malaria infections does not reach a minimum until after five years (61% lower than in 2009).

If the MDA or MSAT is carried out in the peak transmission season for malaria, the maximum decrease in the number of artemisinin-resistant infections is half that which can be achieved in the low transmission season. The frequency of MSAT and MDA was varied and it was found that if carried out twice a year then elimination became possible. If MSAT with AP was undertaken at a maximum of four times a year (it takes three months to complete one round) then elimination can be achieved in eight years. All this is predicated upon resistance not emerging to AP.

The addition of primaquine to annual MDA or MSAT (d) reduces the trough in the number of artemisinin-resistant infections by 20% and the total number of malaria infections by 40%. This extra effect is still insufficient to achieve elimination.

Assuming that malaria vectors bite after people are in or near their beds, then ITNs (e) have a relatively large effect and accelerate the eradication of resistance. The longer that nets are used for, the larger the effect; for example, if the effect of bed nets lasts around four years (the equivalent of long-lasting treated nets) and they reduce transmission by 30%, then time to eradication is reduced by about 50%. Thus a modest, but sustained, protective effect from bed nets or other transmission blocking methods can have a significant impact on the time to elimination.

When a fitness cost of artemisinin resistance is included in the model, the rate of increase of resistant infections is slower (around 33% slower for fitness cost of 5%) and the rate of elimination by an intervention is marginally faster (19% for a fitness cost of 5%). The fitness cost must be less than 7.5% for the number of artemisinin-resistant infections to increase over time.

### Sensitivity analysis

For all interventions, the following were varied in turn: the coverage with ACT from 0 to 100%, the effectiveness of dihydroartemisinin and piperaquine against resistant infections and the cost of piperaquine resistance between 0 and 100%, the cost of artemisinin resistance in terms of fitness between 0 and 5%, the average time to receive anti-malarials during an intervention from 7 to 90 days, and time to natural recovery from infection (i.e. without treatment) from 60 to 200 days. Duration of drug effect was also varied for atovaquone from 10–15 days and for piperaquine from 20–30 days.

The model was most sensitive to ACT coverage, effectiveness of artemisinins against resistant infections, fitness cost of ACT resistance, and time to receive anti-malarials. There was a threshold coverage with ACT of 47% below which the time to eradication was more than a decade. With ACT coverage of <28%, elimination was impossible. The effectiveness of artemisinins against resistant infections was determined from field data to reflect current phenotypes [7] but it is likely that this will decrease over time with continued use of artemisinins. The model predicts that if this effectiveness halves compared to 2009, time to eradication will take 50% longer. If time to receive anti-malarials (ACT or AP) doubles (from 16 to 32 days after developing blood stage infection) time to elimination increases more than threefold, whereas if people receive anti-malarials in half the time (eight days after blood stage infection), time to elimination is three times less. The time to eradication was unaffected by natural recovery rate from infection, and the effectiveness of and fitness cost of resistance to piperaquine. Changing the duration of atovaquone and piperaquine effect had minimal impact on the results. To explore the effect of synergy between the components of ACT, the rate of clearance of infection was varied from 1/3 days^-1 ^to 1/7 days^-1^. This had negligible effect on the time to elimination but decreasing the clearance rate of gametocytes by ACT to 1/7 days^-1 ^decreased the percentage of artemisinin-resistant infections at the time of elimination from 82% to 54%.

## Discussion

Using a relatively simple modeling framework, it was shown that eliminating the use of artemisinin monotherapy and replacing it with ACT can be sufficient to eradicate artemisinin-resistant malaria in Cambodia. Short-term or pulsed interventions such as MSAT or MDA are not sufficiently effective to achieve eradication on their own. Their additional effect, when added to an elimination of monotherapy and replacement with ACT, is so small that it is questionable whether they should be considered at all. AP is more effective than ACT when used in MSAT or MDA but the additional impact of MSAT or MDA, when used in addition to elimination of artemisinin monotherapy and switching treatment to ACT, is small. AP also has the disadvantages of slow clinical responses, high purchase cost, and a low threshold for high-grade resistance and is not therefore being considered for routine first-line treatment of symptomatic cases. In stark contrast, ITNs greatly accelerate the impact of ACT, provided they are effective. Some vectors in this region bite very early in the evening and where these predominate, ITNs would have marginal benefits. Assuming they are effective the ideal combination appears to be long-lasting insecticide-treated bed nets and high coverage with ACT treatment.

If ACTs are used to eliminate artemisinin-resistant malaria then these models provide an important caveat. Elimination of artemisinin-resistant malaria requires the elimination of all malaria as the last few infections to be cleared are almost all resistant. ACT provides a selective pressure for artemisinin resistance, especially in infections with concomitant resistance to the partner drug (piperaquine). The result of this is that ACT produces an increase in the proportion of infections resistant to artemisinin every year that it is used. Monitoring studies would indicate an increase in resistance as incidence and prevalence fell. Any intervention aimed at achieving elimination must, therefore, be used continually through to elimination as discontinuing its use too early would allow the number of infections to increase again but this time a higher proportion of resistant infections would be present than if no intervention had been attempted.

## Conclusion

The model presented here assumes that resistance to artemisinin monotherapy has already emerged on the Thai-Cambodian border. Early intervention has a greater chance of preventing its spread than delaying until higher levels of resistance develop (which would be more difficult to contain). Even if resistance is not present, eliminating artemisinin monotherapies and replacing them with an effective ACT with high coverage should remove the potential threat of emergence and spread of resistance, thus increasing the effective life span of this important class of anti-malarial drugs worldwide. This modeling exercise provides some general guidelines and principles on elimination of artemisinin resistance using ACT, AP, primaquine and ITNs. In summary, rapid, efficient and sustained action could combat the significant risk that artemisinin resistance poses to global public health. Attacking the last man standing is a bold strategy since failure could result in a far more resistant population than existed before the intervention. This strategy, therefore, requires a steadfast commitment from donors to ensure success.

## Competing interests

The authors declare that they have no competing interests.

## Authors' contributions

RJM created the mathematical models and wrote the paper. WP-N, SS and RA assisted with model design and development. LJW supervised the work. NPJD, NJW, SY and AMD contributed to the data input and model parameters and reviewed the manuscript.

## Supplementary Material

Additional File 1 **Supplementary Information**. Summary equations, assumptions and parameters. Click here for file

Additional File 2 **Full Model Code**. Full model code plus instructions for running in Berkeley Madonna™. Click here for file
