# Supplementary material for: The last man standing is the most resistant: eliminating artemisinin-resistant malaria in Cambodia
Source: Malar J. 2009 Feb 20;8:31. doi: 10.1186/1475-2875-8-31 (PMC2660356; doi:10.1186/1475-2875-8-31)
Supplement: Additional File 1 — Supplementary Information. Summary equations, assumptions and parameters. [file 1475-2875-8-31-S1.pdf]

Supplementary Online Material

Summary Equations

The system is described by a set of nonlinear ordinary differential equations of the form:

$$
\begin{aligned}
\dot{S}_{dg} &= b_d N - \frac{(1-\rho)\beta S_{dg} \sum_{g\in G}\sum_{d\in D}\sum_{r\in R}(1-c_r) I_{rdg}}{N} + \sum_{r\in R}\left(\nu_{L,rdg}L_{rdg} + \nu_{B,rdg}B_{rdg} + \nu_{I,rdg}I_{rdg}\right) - \mu S_{dg} + \mathbf{Y}_{\mathbf{S}\,d} + f_S(\boldsymbol{\tau},\mathbf{S},\mathbf{L},\mathbf{B},\mathbf{I}) \\
\dot{L}_{rdg} &= \frac{(1-\rho)\beta S_{dg} \sum_{g\in G}\sum_{d\in D}(1-c_r) I_{rdg}}{N} - \left(\gamma + \nu_{L,rdg} + \mu\right)L_{rdg} + \mathbf{Y}_{\mathbf{L}\,d} + f_L(\boldsymbol{\tau},\mathbf{S},\mathbf{L},\mathbf{B},\mathbf{I}) \\
\dot{B}_{rdg} &= \gamma L_{rdg} - \left(\sigma + \nu_{B,rdg} + \mu\right)B_{rdg} + \mathbf{Y}_{\mathbf{B}\,d} + f_B(\boldsymbol{\tau},\mathbf{S},\mathbf{L},\mathbf{B},\mathbf{I}) \\
\dot{I}_{rdg} &= \sigma B_{rdg} - \left(\nu_{I,rdg} + \mu\right)I_{rdg} + \mathbf{Y}_{\mathbf{I}\,d} + f_I(\boldsymbol{\tau},\mathbf{S},\mathbf{L},\mathbf{B},\mathbf{I})
\end{aligned}
$$

$$
\begin{aligned}
& r \in R = \{none, a, b\}, \quad d \in D = \{none, a, b, ab\}, \quad g \in G = \{0,1,2,...\} \\
& \mathbf{b} = \begin{pmatrix}\mu & 0 & 0 & 0\end{pmatrix} \\
& \mathbf{c} = \begin{pmatrix}0 & c_a & c_b\end{pmatrix}
\end{aligned}
$$

$$
\begin{aligned}
X_{none} &= \begin{pmatrix} 0 & \frac{1}{x_a} & \frac{1}{x_b - x_a} & 0 \end{pmatrix} \\
X_a &= \begin{pmatrix} 0 & -\frac{1}{x_a} & 0 & 0 \end{pmatrix} \\
X_b &= \begin{pmatrix} 0 & 0 & -\frac{1}{x_b - x_a} & \frac{1}{x_a} \end{pmatrix}, \\
X_{ab} &= \begin{pmatrix} 0 & 0 & 0 & \frac{1}{x_a} \end{pmatrix}
\end{aligned}
$$

$$
\begin{aligned}
\mathbf{S_g} &= \begin{pmatrix} S_{none\ g} & S_{a\ g} & S_{b\ g} & S_{ab\ g} \end{pmatrix} & \mathbf{Y_{S\,d}} &= \mathbf{X_d} \bullet \mathbf{S_g}^T \\
\mathbf{L_{rg}} &= \begin{pmatrix} L_{r\ none\ g} & L_{r\ a\ g} & L_{r\ b\ g} & L_{r\ ab\ g} \end{pmatrix} & \mathbf{Y_{L\,d}} &= \mathbf{X_d} \bullet \mathbf{L_{rg}}^T \\
\mathbf{B_{rg}} &= \begin{pmatrix} B_{r\ none\ g} & B_{r\ a\ g} & B_{r\ b\ g} & B_{r\ ab\ g} \end{pmatrix} & \mathbf{Y_{B\,d}} &= \mathbf{X_d} \bullet \mathbf{B_{rg}}^T \\
\mathbf{I_{rg}} &= \begin{pmatrix} I_{r\ none\ g} & I_{r\ a\ g} & I_{r\ b\ g} & I_{r\ ab\ g} \end{pmatrix} & \mathbf{Y_{I\,d}} &= \mathbf{X_d} \bullet \mathbf{I_{rg}}^T
\end{aligned}
$$

Artemisinin is represented as a and piperaquine as b with ACT being ab.

The sets R, D and G refer to the categories of resistance, drug activity and intervention strategy respectively. The arrays ν (recovery from infection under the action of the drugs i.e. recovery rates), τ (rate of drug acquisition i.e. treatment rates) and f (intervention treatment strategies) depend on the nature of the intervention strategies and combinations of drugs.

The arrays **X** and **Y** define the dynamics of the sequential loss of drug effect where x_a is the duration of ACT treatment plus the time post treatment to sub therapeutic levels of artesunate and x_b is the duration of ACT treatment plus the time post treatment to sub therapeutic levels of the partner drug. *T* represents the transpose function on the associated vector.

This deterministic model was also rewritten as two stochastic models; one population based and another individual based. All three models used the same structure and parameters. The population dynamic stochastic model was a set of difference equations based on the Euler approximation of the corresponding differential equations as the means of a set of Poisson distributions from which the value of each variable was sampled at each time step. For the individual based model, a population of individuals was generated with a list of states which defined the variables of the corresponding deterministic model. The individuals change from one state to another with probabilities defined by the parameters of the corresponding deterministic model apart from the transmission parameter. In this instance, transition from uninfected to blood stage was modeled as the probability of a susceptible individual receiving an infectious bite from a mosquito which had previously bitten an infected individual chosen at random. These two stochastic models produced very similar results and 200 runs of the population based model were used to generate the results given in the paper.

Supplementary tables

Table S1. Assumptions.

Assumption	Reasoning for likely effect on time to eradication if true	Justification for making the assumption
**Likely to *increase* time to eradication of artesunate resistance***		
No immunity to malaria i.e. transmission rate is low.	Infection more likely to result in symptoms therefore more people seeking and receiving treatment.	Transmission rates in Western Cambodia are generally much lower than in sub-Saharan Africa, for example [1]. There are small focal areas with higher rates and we plan to explore this with a spatially heterogeneous model when sufficient data becomes available.
Low (0 to 5%) survival disadvantage (fitness cost) for artesunate resistant parasites compared to drug sensitive parasites.	More robust resistant parasites are harder to eradicate.	The relative viability of malaria parasites with artesunate resistant phenotypes is not known, although survival disadvantage, if it exists at all, is likely to be minimal [2].
No mortality due to malaria.	Those people with resistant infections are less likely to respond to treatment and therefore more likely to die, thus removing them from the transmitting population.	In reality the proportion of malaria infections which are fatal in this region is low, around 0.6% [3]
Artesunate is the only available effective treatment before 2009.	Artesunate would be less likely to cure artemisinin-resistant infections than non-artemisinin drugs, if	Although co-blistered artesunate and mefloquine has been the official first-line drug since 2000, in reality a wide range of

	available.	treatments is available over the counter in Cambodia. The majority receive artesunate monotherapy whereas most of the other treatments are inadequate (resistant parasites/wrong dose/wrong duration) to cure infection [4].
Rate of resistance to artesunate is increasing exponentially at the time of intervention.	If the rate of resistance was stable or decreasing then infection would be easier to eradicate.	Expert opinion in the absence of historical data.
Rate of resistance to piperaquine is stable at the time of intervention.	If the rate of piperaquine resistance was increasing then piperaquine would take longer to eradicate infection.	Expert opinion in the absence of historical data.
**Likely to *decrease* time to eradication of artesunate resistance***		
No pre-existing resistance to DHA / piperaquine ACT, only to artesunate and piperaquine alone.	If there are infections resistant to ACT then these will be even harder to cure than those with resistance to the individual drugs.	No evidence for pre-existing resistance to ACT has ever been found.
No resistance to atovaquone, proguanil or primaquine.	Interventions using these drugs will have maximal effectiveness if there is no resistance.	Rates of resistance to each of these drugs are thought to be low in this region.
Recombination between drug resistant mutants not frequent enough to have a significant effect	Recombination has the potential to generate parasites resistant to both components of ACT.	There is no strong evidence for frequent recombination combining drug resistance mutations in malaria.

in the model timescale.		The genetics of resistance to artemisinins and piperaquine are unknown therefore a model of this would be conjectural<br><br>If the inheritance of resistance to either of these drugs is polygenic, e.g. acquired incrementally by the acquisition of a series of mutations, then recombination would decrease the strength and prevalence of resistance.
No spatial heterogeneity i.e. transmission, coverage of interventions, access to health services, etc.	Infection in high transmission areas is harder to eradicate therefore taking longer.	This is potentially important for planning interventions but it is not known to what degree this exists in Western Cambodia.<br><br>In the absence of data about most spatially heterogeneous parameters we felt their incorporation at this stage was premature.<br><br>We are in the ongoing process of gathering data to allow the incorporation of realistic spatial heterogeneity into the next stage of this model.
No migration.	People do not continue to introduce new resistant parasites.	To maintain simplicity.<br><br>In-migration of sensitive infections would accelerate the elimination of resistance. In-

		migration of resistant infections would slow it. Out-migration of resistant infections would mean control/elimination efforts would have to include these areas also in order to achieve elimination.<br><br>As we are modeling containment strategies for the only area where artesunate resistance has been identified, Inmigration of resistant infections is not relevant.

* The first 6 assumptions are all likely to increase the time to eradication of resistance, whereas the other 5 assumptions probably decrease the time to eradication, for the reasons given. Hence this model is probably conservative overall.

Table S2. Parameters. These were based largely on expert opinion of the co-authors and were derived from published data, where available, as stated below. For those parameters for which a range of values is given, this reflects uncertainty of their true value. For these parameters, the underlined values were used to generate the plots and results stated in the text and the ranges were used in the sensitivity analysis.

Symbol	Description	Value	Source
**Population demographics**			
N_0	Total population size	$3.2*10^6$	[5]
μ	Birth rate = death rate	15/1000/year	[6]
	**Prevalence of malaria in population**		
p_{Bl}	Proportion of population with slide positive malaria infection in high transmission season in 2009	0.074	[1]
p_{inf}	Proportion of population with infectious blood stage infection at time=0	0.16	(the value required to give pBI ~ 0.074)
p_a	Proportion of malaria infections that are resistant to artesunate in 2008	0.1	Expert opinion
p_b	Proportion of malaria infections that are	0.05	Expert opinion

	resistant to piperaquine in 2009		
**Natural history of malaria infection**			
δ	Natural recovery rate from infection	1/200 - 1/60 days^{-1}	[7-12]
γ	Rate of liver stage becoming blood stage	1/5 days^{-1}	[7-9]
σ	Rate of blood stage becoming gametocytes	1/15 days^{-1}	[13,14]
amp	Amplitude of seasonal variation of transmission	0.67	[15]
**Rates of initiation and proportions of population receiving drug treatment**			
	**Artemisinin monotherapy**		
$start_a$	Year of introduction of artemisinin monotherapy	1975	Expert opinion
$\square = \square_{ai1} = \square_{ai2}$	Rate of starting artemisinin monotherapy	1/16 infected people per day	[4]
$propRx_{am}$	Proportion of infected population who receive antimalarials	0.63	[4]
$prop_a$	Proportion of antimalarials constituting artemisinin monotherapy before an	0.4	[4]

	intervention		
adh_a	Proportion of infected population that take full 7 day course of artemisinin monotherapy	0.2	[4]
$propRx_a$	Proportion of infected population that take effective artemisinin monotherapy = propRxam*propa*adha	0.05	= $propRx_{am}*prop_a*adh_a$
	**Interventions**		
$\square_{ab} = \square_{ab1} = \square_{ab2}$	Rate of starting ACT for treatment	16 infected people per day	[4]
$\square_1 = \square_2 = \square_3$	Rate of reaching maximum coverage for MDA or MSAT	1/0.25 $years^{-1}$	Expert opinion
$cov_{i1} = cov_{i2} = cov_{i3}$	Maximum coverage of MDA or MSAT	0.8	Expert opinion
cov_{ab}	Maximum coverage with ACT after replacement of artemisinin monotherapy	0.6	Expert opinion
p_{sab}	Proportion of vendors selling modern drugs that could sell ACT	0.85	[4]

adh_{ab} = adh_{vg}	Adherence to 3 day course of ACT = Adherence to 3 days of atovaquone/ proguanil	0.77	[4]
$propRx_{i1}$ = $propRx_{i2}$ = $propRx_{i3}$	Proportion that receive full 3 day course of MDA/MSAT	0.616	= $cov_{i1}*adh_{ab}$ or $cov_{i2}*adh_{vg}$ or $cov_{i3}*adh_{ab}$
p_{ab}	Proportion that receive full 3 day course of ACT after switch	0.3927	=$cov_{ab}*p_{sab}*adh_{ab}$
**Duration of intervention and drug availability**			
dur_1 = dur_2 = dur_3	Total duration of MDA or MSAT	0 years – long term	Expert opinion
n_{i2}	Number of times per year MSAT with atovaquone/proguanil is carried out	1-4 $years^{-1}$	Expert opinion
$dur_{\tau1}$ = $dur_{\tau2}$ = $dur_{\tau3}$	Duration of each pulse of MDA or MSAT	0.25 years	Expert opinion
dur_a	Duration of availability of artemisinin	0 years or long-term	Expert opinion

	monotherapy		
dur_{ab}	Duration of availability of ACT	0 years or long-term	Expert opinion
dur_{bn}	Duration of effectiveness of bed nets	0 or 4 years	Expert opinion
**Drug pharmacodynamics**			
	**Duration of efficacy against sensitive parasites (X)**		
X_{ao}	Full course of artemisinin monotherapy	7 days	[16]
X_{ai}	Dihydroartemisinin as part of ACT (3 day course)	3 days	[16]
X_b	Piperaquine	20-30 days	[17]
X_v	Atovaquone (as 3 days atovaquone/proguanil)	10-15 days	[18]
X_g	Proguanil (as 3 days atovaquone/proguanil)	4 days	[19]
X_p	Primaquine (1 day course)	1 day	[20]
	**Rates of clearance of drug sensitive infection (ν) by treatment**		

c_{Broda}	Artemisinin vs noninfectious blood stage	1/7 $days^{-1}$	[21]
c_{Iroda}	Artemisinin vs infectious blood stage	1/4$days^{-1}$	Unpublished data from Lee S
c_{Brodb}	Piperaquine vs noninfectious blood stage	1/3 $days^{-1}$	[22]
c_{Irodb}	Piperaquine vs infectious blood stage	1/21 $days^{-1}$	[22]
c_{rodab}	ACT vs any stage	1/7 $days^{-1}$ (no synergy assumed) – 1/3 $days^{-1}$ (synergy assumed)	[21,23,24]
c_{Ldvg}	Atovaquone/proguanil vs liver stage	1/3 $days^{-1}$	[25]
c_{Bdvg}	Atovaquone/proguanil vs non-infectious blood stage	1/3 $days^{-1}$	[18]
$c_{Idvg} = c_{Idv}$	Atovaquone/proguanil vs infectious blood stage= atovaquone vs infectious blood stage	1/(4.5) $days^{-1}$	Unpublished data from Lee S
c_{Ldv}	Atovaquone vs liver stage	1/6 $days^{-1}$	[25]
c_{Bdv}	Atovaquone vs non-infectious blood stage	1/3 $days^{-1}$	[18]

	infection		
c_{Ldp}	Primaquine vs liver stage infection	1/7days^{-1}	[18]
c_{Idp}	Primaquine vs infectious blood stage infection	1/1 days^{-1}	[20,26]
	**Effect of drug resistance on pharmacodynamics** As this is unknown, it was a modeled by multiplying the clearance rate for each drug by its relative effectiveness against resistant infections, ε, such that $0 \leq \varepsilon \leq 1$.		
pct_{roda}	Parasite clearance time for artemisinin vs sensitive infections	30 hours	[27]
pct_{rada}	Parasite clearance time for artemisinin vs resistant infections	83 hours	[28]
p_{recra}	Proportion of infections resistant to artemisinin that recrudesce after treatment with artemisinin monotherapy	0.35	[28]
ε_{rada}	Relative effectiveness of artemisinin against artemisinin resistant parasites	0.27	$= pct_{roda}/pct_{rada}*(1-p_{recra})$
ε_{rbdb}	Relative effectiveness of piperaquine against resistant parasites	0.8	[22]

cost	**Fitness cost of drug resistance**<br>This was modeled by multiplying the transmission parameter β by (1-cost) [29] for each drug:		
$cost_a$	Artemisinin	0 - 0.1	[2]
$cost_b$	Piperaquine	**0** - 0.1	[2]
**Effectiveness of bednets**			
ρ	Degree of transmission reduction (the product of coverage and efficacy)	0.3	[30,31]

References for Supplementary Online Material

1. Incardona S, Vong S, Chiv L, Lim P, Nhem S, Sem R, Khim N, Doung S, Mercereau-Puijalon O, Fandeur T: **Large-scale malaria survey in Cambodia: novel insights on species distribution and risk factors.** *Malaria J* 2007, **6:**37.
2. Walliker D, Hunt P, Hamza Babiker H: **Fitness of drug-resistant malaria parasites.** Acta Trop 2005, **94:**251-259.
3. **Cambodia Health Situation and Trend.** World Health Organization Regional Office for the Western Pacific; 2007. [http://www.wpro.who.int/NR/rdonlyres/BED34D62-D904-43B7-9CEC-ADA3CE991459/0/8Cambodia07.pdf]
4. Yeung S, Van Damme W, Socheat D, White NJ, Mills A: **Access to artemisinin combination therapy for malaria in remote areas of Cambodia.** Malaria J 2008, **7:**96.

5. **First revision populations for Cambodia 1998-2020.** National Institute of Statistics of Cambodia, Ministry Of Planning; 1998. [http://statsnis.org/projcam/Provinfo_Proj.htm]

6. **Cambodia Inter-Censal Population Survey.** National Institute of Statistics of Cambodia, Ministry Of Planning; 2004. [http://statsnis.org/SURVEYS/depth-cips04/pro-cips/Table3_projection.htm]

7. Eyles DE, Young MD: **The duration of untreated or inadequately treated Plasmodium falciparum infections in the human host.** J Natl Malar Soc 1951, **10:**327-336.

8. Collins WE, Jeffery GM: **A retrospective examination of sporozoite- and trophozoite-induced infections with Plasmodium falciparum: development of parasitologic and clinical immunity during primary infection.** *Am J Trop Med Hyg* 1999, **61:**4-19.

9. Kitchen SF: **Falciparum Malaria.** In *Malariology*. Edited by Boyd MF. Philadelphia: W.B. Saunders Co.; 1949:995-1016.

10. Franks S, Koram KA, Wagner GE, Tetteh K, McGuinness D: **Frequent and persistent, asymptomatic Plasmodium falciparum infections in African infants, characterized by multilocus genotyping.** *J Infect Dis* 2001, **183:**796-804.

11. Bruce MC, Donnelly CA, Packer M, Lagog M, Gobson N: **Age- and species-specific duration of infection in asymptomatic malaria infections in Papua New Guinea.** *Parasitology* 2000, **121:**247-256.

12. Babiker HA, Abdel-Muhsin AM, Ranford-Cartwright LC, Satti G, Walliker D: **Characteristics of Plasmodium falciparum parasites that survive the lengthy dry season in eastern Sudan where malaria transmission in markedly seasonal.** *Am J Trop Med Hyg* 1998 **59:**582-590.

13. Thompson D: **A research into the production, life and death of crescents in malignant tertian malaria, in treated and untreated cases by an enumerative method.** *Ann Trop Med Parasitol* 1911, **5:**57-85.

14. Jeffrey GM, Eyles DE: **Infectivity to mosquitoes of Plasmodium falciparum as related to gametocyte density and duration of infection.** *Am J Trop Med Hyg* 1955, **4:**781-789.

15. Ministry of Health National Center for Parasitology, Entomology and Malaria Control, Kingdom of Cambodia: *Annual progress report of the National Center for Parasitology, Entomology and Malaria Control.* Phnom Penh; 2007:26-39.

16. White NJ: **Assessment of the pharmacodynamic properties of antimalarial drugs in vivo.** *Antimicrob Ag Chemother* 1997, **41:**1413-1422.

17. Tarning J, Ashley EA, Lindegardh N, Stepniewska K, Phaiphun L, Day NP, McGready R, Ashton M, Nosten F, White NJ: **Population pharmacokinetics of piperaquine after two different treatment regimens with dihydroartemisinin-piperaquine in patients with Plasmodium falciparum malaria in Thailand.** *Antimicrob Ag Chemother* 2008, **52:**1052-1061.

18. van Vugt M, Leonardi E, Phaipun L, Slight T, Thway KL, McGready R, Brockman A, Villegas L, Looareesuwan S, White NJ, Nosten F: **Treatment of uncomplicated multidrug-resistant falciparum malaria with artesunate-atovaquone-proguanil.** *Clin Infect Dis* 2002, **35:**1498-1504.

19. Wattanagoon Y, Taylor RB, Moody RR, Ochekpe NA, Looareesuwan S, White NJ: **Single dose pharmacokinetics of proguanil and its metabolites in healthy subjects.** *Br J Clin Pharmacol* 1987, 24: 775-780.

20. Burges RW, Bray RS: **The effect of a single dose of primaquine on the gametocytes, gametogony and sporogony of Laverania falciparum.** *Bull World Health Organ* 1961, 24: 451-456.

21. International Artemisinin Study Group: **Artesunate combinations for treatment of malaria: meta-analysis.** Lancet 2004, **363:**9-17.

22. Chen L, Qu FY, Zhou YC: **Field observations on the antimalarial piperaquine.** *Chin Med J* 1982, **95:**281-286.

23. Myinta HY, Ashley EA, Day NJP, Nosten F, White NJ: **Efficacy and safety of dihydroartemisinin-piperaquine.** *Trans R Soc Trop Med Hyg* 2007, **101:**858-866.

24. Janssens B, van Herp M, Goubert L, Chan S, Uong S, Nong S, Socheat D, Brockman A, Ashley EA, Van Damme W: **A randomized open study to assess the efficacy and tolerability of dihydroartemisinin-piperaquine for the treatment of uncomplicated falciparum malaria in Cambodia.** *Trop Med Int Health* 2007, **12:**251-259.

25. Berman JD, Nielsen R, Chulay JD, Dowler M, Kain KC, Kester KE, Williams J, Whelen AC, Schmuklarsky MJ: **Causal prophylactic efficacy of atovaquone-proguanil (Malarone) in a human challenge model.** *Trans R Soc Trop Med Hyg* 2001, **95:**429-432.

26. Rieckman KH, McNamara JV, Kass L, Powell RD: **Gametocytocidal and Sportontocidal Effects of Primaquine Upon Two Strains of Plasmodium Falciparum.** *Mil Med* 1969, **134:**802-819.

27. Karbwang J, Na-Bangchang K, Congpoung K, Thanavibul A, Harinasuta T: **Pharmacokinetics of oral artesunate in thai patients with uncomplicated falciparum malaria.** *Clin Drug Investig* 1998, **15:**37-43.

28. Dondorp AM, Nosten F, Poravuth, Das D, Phae Phyo A, Tarning J, Lwin KM, Ariey F, Hanpithakpong W, Lee SJ, Ringwald P, Silamut K, Imwong M, Chotivanich K, Pharath L, Herdman T, An SS, Yeung S, Singhasivanon P, Day NPJ, Lindegardh N, Socheat D, White NJ: **Reduced *in-vivo* susceptibility of *Plasmodium falciparum* to artesunate in Western Cambodia.** *N Engl J Med*, in press.

29. Boni MF, Smith DL, Laxminarayan R: **Benefits of using multiple first-line therapies against malaria.** *Proc Natl Acad Sci USA* 2008, **105:**14216-14221.

30. Dolan G, ter Kuile FO, Jacoutot V, White NJ, Luxemburger C, Malankirii L, Chongsuphajaisiddhi T, Nosten F: **Bed nets for the prevention of malaria and anaemia in pregnancy.** *Trans R Soc Trop Med Hyg* 1993, **87:**620-626 .

31. Sochantha T, Hewitt S, Nguon C, Okell L, Alexander N, Yeung S, Vannara H, Rowland M, Socheat D: **Insecticide-treated bednets for the prevention of Plasmodium falciparum malaria in Cambodia: a cluster-randomized trial.** *Trop Med Int Health* 2006, **11:**1166-1177.
