# Supplementary material for: The last man standing is the most resistant: eliminating artemisinin-resistant malaria in Cambodia
Source: Malar J. 2009 Feb 20;8:31. doi: 10.1186/1475-2875-8-31 (PMC2660356; doi:10.1186/1475-2875-8-31)
Supplement: Additional File 2 — Full Model Code. Full model code plus instructions for running in Berkeley Madonna™. [file 1475-2875-8-31-S2.pdf]

**Full Model Code**

The model used in this paper was created using a software package called Berkeley Madonna™. (Berkeley Madonna™ can be downloaded as a free trial version at http://www.berkeleymadonna.com/download.html)

Below is the full Berkeley Madonna™ code for the model.

In order to run the model, the following steps should be followed:

1) Replace the terms β, γ, δ, μ, π, ρ, σ and τ with their names e.g. beta, gamma, etc.
2) Cut and paste the entire code into the Equations window of Berkeley Madonna™
3) Run the code with $T_{BI}$ (total number of blood stage infections), $N_{ra}$ (number of infections resistant to artesunate) and $perc_{ra}$ (percentage of infections resistant to artesunate) displayed in the Graph window
4) Use the parameter sliders to alter parameter values to explore their effects on the results

```
STARTTIME = 1960
STOPTIME=2050
DT = 0.0027

;Model to examine the effect of elimination interventions on artesunate resistance in Western Cambodia
;Dr Richard Maude 27/11/08

;All times are in years
;Artesunate monotherapy begins in 1975
;Artesunate resistance first arises in 1980
;Interventions begin in 2009

;NOTATION
;N = total population size (N = S+L+B+I)
;t = time for drug to clear sensitive infection
;c = clearance rate of infection by drug according to drug sensitivity
;S = susceptible people
;L = liver stage infections
;B = noninfectious blood stage infections
;T = total
;I = infectious blood stage infections
;r = resistant to...
;d = drug given
;o = no drug
;a = artesunate
;ai = artesunate as part of ACT
```

;b = piperaquine
;ab = ACT (dihydroartemisinin plus piperaquine)
;p = primaquine

;e.g.1 $t_{Bda}$ = time for artesunate to clear noninfectious blood stage infection
;e.g.2 $c_{Bradab}$ = clearance rate (c) of noninfectious blood stage infections (B) resistant to artesunate (ra) by ACT (dab)
;e.g.3 $c_{Irodp}$ = clearance rate (c) of infectious blood stage infections (I) resistant to no drugs i.e. sensitive to all (ro) by primaquine (dp)

;$i_o$ = no intervention
;$i_1$ = mass drug administration (MDA) (Intervention 1)
;$i_{1a}$ = treatment of symptomatic patients who have already received MDA
;$i_2$ = masss screen and treat (MSAT) with atovaquone/proguanil (Intervention 2)
;$i_3$ = MSAT with ACT (Intervention 3)

;e.g.4 $d_{ai1a}$ = treatment of symptomatic patients with artesunate monotherapy ($d_a$) who have already received MDA
;e.g.5 $d_{abi1a}$ = treatment of symptomatic patients with ACT who have already received MDA
;X = effective duration of drug action
;e.g.6 $X_{ai}$ = effective duration of artesunate as part of ACT

;e = relative effectiveness of drug against drug resistant infections vs drug sensitive infections
;bn = bed nets
;dur = duration

;PARAMETERS

;Population parameters
$\mu$ = 0.015 ; i.e. birth rate & death rate
$N_0$ = 3200000 ; total population at time=0
$I_0 = N_0 * p_{inf}$ ; total infected at time=0

;Natural history of infection
$\gamma$ = 365/5 ; rate of liver stage becoming noninfectious blood stage
$\sigma$ = 365/15 ; rate of developing gametocytes
$\delta$ = 365/60 ; natural recovery rate of infectious

;Drug action

;Time for drug to clear drug sensitive infection in days

$t_{Bda}$ = 7 ; time for artesunate to clear B
$t_{Ida}$ = 4 ; time for artesunate to clear I
$t_{Bdb}$ = 3 ; time for clearance of noninfectious blood stage by piperaquine
$t_{Idb}$ = 21 ; time for clearance of infectious blood stage by piperaquine
$t_{Bdab}$ = 3 ; time for ACT to clear B = 3 days if synergy or 7 days if none
$t_{Idab}$ = 3 ; time for ACT to clear I = 3 days if synergy or 4 days if none
$t_{Ldvg}$ = 3 ; time for clearance of liver stage by atovaquone/proguanil
$t_{Bdvg}$ = 3 ; time for clearance of noninfectious blood stage by atovaquone/proguanil
$t_{Idvg}$ = 4.5 ; time for clearance of infectious blood stage by atovaquone/proguanil
$t_{Ldv}$ = 6 ; time for clearance of liver stage by atovaquone
$t_{Bdv}$ = 3 ; time for clearance of noninfectious blood stage by atovaquone
$t_{Idv}$ = 4.5 ; time for clearance of infectious blood stage by atovaquone
$t_{Ldp}$ = 7 ; time for clearance of liver stage by primaquine
$t_{Idp}$ = 1 ; time for clearance of infectious blood stage by primaquine

;rate of clearance of infection by drug according to drug sensitivity
$c_{Brodo}$ = 0 ; rate of clearance of sensitive noninfectious blood stage by no drug
$c_{Brado}$ = 0 ; rate of clearance of artesunate resistant noninfectious blood stage by no drug
$c_{Brbdo}$ = 0 ; rate of clearance of piperaquine resistant noninfectious blood stage by no drug
$c_{Irodo}$ = 0 ; rate of clearance of sensitive infectious blood stage by no drug
$c_{Irado}$ = 0 ; rate of clearance of artesunate resistant infectious blood stage by no drug
$c_{Irbdo}$ = 0 ; rate of clearance of piperaquine resistant infectious blood stage by no drug
$c_{Broda} = 365/t_{Bda}$ ; rate of clearance of blood stage resistant to none treated with artesunate
$c_{Brada} = e_{rada}*c_{Broda}$ ; rate of clearance of blood stage resistant to artesunate treated with artesunate
$c_{Brbda} = 365/t_{Bda}$ ; rate of clearance of blood stage resistant to piperaquine treated with artesunate
$c_{Iroda} = 365/t_{Ida}$ ; rate of clearance of I resistant to none treated with artesunate
$c_{Irada} = e_{rada}*c_{Iroda}$ ; rate of clearance of I resistant to artesunate treated with artesunate
$c_{Irbda} = 365/t_{Ida}$ ; rate of clearance of I resistant to piperaquine treated with artesunate
$c_{Brodab} = 365/t_{Bdab}$ ; rate of clearance of B resistant to none treated by ACT
$c_{Bradab} = e_{rada}*365/t_{Bdab} + (1-e_{rada})*365/t_{Bdb}$ ; rate of clearance of artesunate resistant noninfectious blood stage by ACT
$c_{Brbdab} = e_{rbdb}*365/t_{Bdab} + (1-e_{rbdb})*365/t_{Bda}$ ; rate of clearance of piperaquine resistant noninfectious blood stage by ACT
$c_{Irodab} = 365/t_{Idab}$ ; rate of clearance of sensitive infectious blood stage by ACT
$c_{Iradab} = e_{rada}*365/t_{Idab} + (1-e_{rada})*365/t_{Idb}$ ; rate of clearance of artesunate resistant infectious blood stage by ACT
$c_{Irbdab} = e_{rbdb}*365/t_{Idab} + (1-e_{rbdb})*365/t_{Ida}$ ; rate of clearance of piperaquine resistant infectious blood stage by ACT
$c_{Brodb} = 365/t_{Bdb}$ ; rate of clearance of sensitive noninfectious blood stage treated with piperaquine (after artesunate i.e. minus 3 days)
$c_{Bradb} = 365/t_{Bdb}$ ; rate of clearance of artesunate resistant noninfectious blood stage treated with piperaquine
$c_{Brbdb} = e_{rbdb}*365/t_{Bdb}$ ; rate of clearance of piperaquine resistant noninfectious blood stage treated with piperaquine
$c_{Irodb} = 365/t_{Idb}$ ; rate of clearance of sensitive infectious blood stage treated with piperaquine

$c_{Iradb} = 365/t_{Idb}$ ; rate of clearance of artesunate resistant infectious blood stage treated with piperaquine
$c_{Irbdb} = e_{rbdb}*365/t_{Idb}$ ; rate of clearance of piperaquine resistant infectious blood stage treated with piperaquine
$c_{Lp} = p_p*365/t_{Ldp}$ ; rate of clearance of liver stage treated with primaquine
$c_{Ip} = p_p*365/t_{Idp}$ ; rate of clearance of infectious blood stage treated with primaquine
$c_{Ldvg} = 365/t_{Ldvg}$ ; rate of clearance of liver stage treated with atovaquone/proguanil
$c_{Bdvg} = 365/t_{Bdvg}$ ; rate of clearance of noninfectious blood stage treated with atovaquone/proguanil
$c_{Idvg} = 365/t_{Idvg}$ ; rate of clearance of infectious blood stage treated with atovaquone/proguanil
$c_{Ldv} = 365/t_{Ldv}$ ; rate of clearance of liver stage treated with atovaquone after atovaquone/proguanil
$c_{Bdv} = 365/t_{Bdv}$ ; rate of clearance of noninfectious blood stage treated with atovaquone after atovaquone/proguanil
$c_{Idv} = 365/t_{Idv}$ ; rate of clearance of infectious blood stage treated with atovaquone after atovaquone/proguanil

;Effective duration of drug action
$X_{ao} = 7/365$ ; effective duration of artesunate monotherapy
$X_{ai} = 3/365$ ; effective duration of artesunate as part of ACT
$X_b = 20/365$ ; effective duration of piperaquine
$X_g = 4/365$ ; effective duration of proguanil as part of atovaquone/proguanil
$X_v = 15/365$ ; effective duration of atovaquone
$X_p = 1/365$ ; effective duration of primaquine

;Drug resistance
$e_{rada} = (1-prec_{ra}) * pct_{roda}/pct_{rada}$ ; relative effectiveness of artesunate against artesunate resistant infections (0-1)
$pct_{roda} = 30$ ; parasite clearance time for artesunate vs sensitive infections
$pct_{rada} = 83$ ; parasite clearance time for artesunate vs resistant infections
$prec_{ra} = 0.35$ ; proportion recrudescences in resistant infections
$e_{rbdb} = 0.8$ ; relative effectiveness of piperaquine in piperaquine resistant infections (0-1)

;Rates of receiving treatments
;Artesunate monotherapy
$\tau = 365/16*propRx_a*SQUAREPULSE(start_a, dur_a)$ ; rate of starting artesunate in infected patients
$dur_a = stop_a-start_a$ ; duration availability of artesunate
$start_a = 1975$ ; time that artesunate first introduced
$stop_a = 2009$ ; time from when artesunate no longer available

;Treat symptomatic infection with ACT
$\tau_{ab} = \tau mag_{ab}*propRx_{ab}*SQUAREPULSE(start_{ab}, dur_{ab})$
$\tau mag_{ab} = 365/16$

$dur_{ab}$ = 41; 'long-term'
$start_{ab}$ = 2009;

;Intervention 1: MDA with ACT
$\tau pulse_1$ = if mod(time-$dur\tau_1$,1)>=1-$dur\tau_1$ then $propRx_{i1}$*$\tau mag_{i1}$ else 0
$\tau_1$= $\tau pulse_1$*SQUAREPULSE(2009, $dur_{i1}$)
$\tau mag_{i1}$ = 4 ; rate of starting intervention 1 in 1/years
$dur_{i1}$ = 0 ; total duration of intervention 1 in years
$dur\tau_1$ = 0.25 ; duration of each round of intervention 1 in years

;Intervention 2: MSAT with atovaquone/proguanil
$\tau pulse_2$ = if mod(time-$dur\tau_2$,$fr_{i2}$)>=$fr_{i2}$-$dur\tau_2$ then $propRx_{i2}$*$\tau mag_{i2}$ else 0
$\tau_2$= $\tau pulse_2$*SQUAREPULSE(2009, $dur_{i2}$)
$\tau mag_{i2}$ = 4 ; rate of starting intervention 2 in 1/years
$dur_{i2}$ = 0 ;  total duration of intervention 2 in years
$dur\tau_2$ = 0.25 ; duration of each round of intervention 2 in years
$fr_{i2}$ = 1/$n_{i2}$;1/frequency of intervention 2 per year
$n_{i2}$ = 1; frequency of intervention 2 per year

;Intervention 3: MSAT with ACT.
;this uses the same section of code as switching treatment to ACT
; to make this run, do the following:
;1) add a ';' to the beginning of the first line of the 'treat symptomatic infection with ACT' section (above) to give ";$\tau_{ab}$ = $\tau mag_{ab}$*$propRx_{ab}$*SQUAREPULSE($start_{ab}$, $dur_{ab}$)"
$\tau pulse_3$ = if mod(time-$dur\tau_3$,1)>=1-$dur\tau_3$ then $propRx_{i3}$*$\tau mag_{i3}$ else 0
;2) remove the ';' from the beginning of the line ";$\tau_{ab}$= $\tau pulse_3$*SQUAREPULSE(2009, $dur_{i3}$)" (below)
$\tau pulse_3$ = if mod(time-$dur\tau_3$,1)>=1-$dur\tau_3$ then $propRx_{i3}$*$\tau mag_{i3}$ else 0
;$\tau_{ab}$= $\tau pulse_3$*SQUAREPULSE(2009, $dur_{i3}$)
$\tau mag_{i3}$ = 4 ; rate of starting intervention 3 in 1/years
$dur_{i3}$ = 0 ; total duration of intervention 3 in years
$dur\tau_3$ = 0.25 ; duration of each round of intervention 3 in years

;ACT for symptomatic infection during intervention 1
$\tau_{abi1}$ = $\tau mag_{abi1}$*$propRx_{ab}$*SQUAREPULSE($start_{ab}$, $dur_{ab}$); rate of starting ACT in infected people during intervention 1
$\tau mag_{abi1}$ = 365/16

;Artesunate monotherapy for symptomatic infection during intervention 1
$\tau_{ai1}$ = $\tau mag_{ai1}$*$propRx_a$*SQUAREPULSE(2009, $dur_a$); rate of starting artesunate in infected people during intervention 1
$\tau mag_{ai1}$ = 365/16

;ACT for symptomatic infection during intervention 2

$\tau_{abi2} = \tau mag_{abi2}*propRx_{ab}*SQUAREPULSE(2009, dur_{ab})$; rate of starting ACT during intervention 2
$\tau mag_{abi2} = 365/16$

;Artesunate monotherapy for symptomatic infection during intervention 2
$\tau_{ai2} = \tau mag_{ai2}*propRx_a*SQUAREPULSE(2009, dur_a)$; rate of starting artesunate in infected people during intervention 2
$\tau mag_{ai2} = 365/16$

;Bed nets
$bn=bnmag*SQUAREPULSE(2009, dur_{bn})$
bnmag=0.3; transmission reduction due to bed nets
$dur_{bn}=0$; 0 or 4 years

;Initial conditions
$p_a = 0$ ; proportion resistant to artesunate in 1960
$p_b = 0.05$ ; proportion resistant to piperaquine in 1960 (and 2009)
$p_o = 1-p_a-p_b$ ; proportion sensitive to artesunate and piperaquine in 1960
$p_{BI} = 0.0743$ ; proportion infected with detectable blood stage infection in 2009 (data is from 2006)
$p_{inf} = 0.16$ ; proportion any malaria infection in 1960
$p_p = 0$ ; proportion given primaquine = 0-1

st = 1980 ; time that artesunate resistance first arises - this is set relatively early to maximise pa in 2009 so near 10%
mst = 350 ;

$propRx_a = propRx_{am}*prop_a*adh_a$ ; proportion that get effective artesunate treatment for symptomatic infection = 0.052
$propRx_{am} = 0.63$ ; proportion receiving antimalarials
$prop_a = 0.4$ ; proportion of antimalarials constituting artesunate monotherapy
$adh_a = 0.2$ ; proportion that take full 7 day course artesunate monotherapy

$propRx_{ab}$ = IF TIME<2009 THEN 0 ELSE $p_{ab}*(cov_{ab}-cov_{ab}*exp(-k*(TIME-2009)))$ ; proportion that get effective ACT treatment for symptomatic infection

$cov_{ab} = 0.6$ ; maximum coverage with ACT for treatment
$p_{sab} = 0.85$ ; proportion of shops that sell modern drugs
$adh_{ab} = 0.77$ ; adherence to 3 day regime of ACT
$adh_{vg} = 0.77$ ; adherence to 3 day regime of atovaquone/proguanil
$p_{ab} = cov_{ab}*p_{sab}*adh_{ab}$ ; proportion that actually take 3 day course of ACT

k = 30 ; speed of introduction of intervention

$propRx_{i1} = cov_{i1}*adh_{ab}$ ; proportion that complete a 3 day course of MDA with ACT
$propRx_{i2} = cov_{i2}*adh_{vg}$ ; proportion that complete a 3 day course of atovaquone/proguanil during MSAT
$propRx_{i3} = cov_{i3}*adh_{ab}$ ; proportion that complete a 3 day course of ACT during MSAT
$cov_{i1} = 0.8$ ; coverage with intervention 1
$cov_{i2} = 0.8$ ; coverage with intervention 2
$cov_{i3} = 0.8$ ; coverage with intervention 3

;Calculate intial conditions

ROOTI $\beta_n = \mu*N_0 - \beta_n*(p_{inf}*N_0)*S_{rodoio1}/N_0+\delta*(p_{inf}*N_0)-\mu*S_{rodoio1}$
ROOTI $S_{rodoio1} = \beta n*(p_{inf}*N_0)*S_{rodoio1}/N_0-(\gamma+\mu)*L_{rodoio1}$
ROOTI $L_{rodoio1} = \gamma*L_{rodoio1}-(\sigma+\mu)*B_{rodoio1}$
ROOTI $B_{rodoio1} = \sigma*B_{rodoio1}-(\delta+\mu)*(p_{inf}*N_0)$

GUESS $\beta_n = 10$
LIMIT $\beta_n >= 0$
LIMIT $\beta_n <= 1000$

GUESS $S_{rodoio1} = 2.1e+6$
LIMIT $S_{rodoio1} >= 0$
LIMIT $S_{rodoio1} <= N0$

GUESS $L_{rodoio1} = 7e+4$
LIMIT $L_{rodoio1} >= 0$
LIMIT $L_{rodoio1} <= N0$

GUESS $B_{rodoio1} = 2e+5$
LIMIT $B_{rodoio1} >= 0$
LIMIT $B_{rodoio1} <= N0$

;MODEL

;Treatment with artesunate monotherapy
;$N_{doio}$ and $N_{daio}$

;Box $N_{doio}$

init $S_{doio} = S_{rodoio1}$
$I_{doio0} = N_0*p_{inf}$

$L_{doio} = L_{rodoio}+L_{radoio}+L_{rbdoio}$
$B_{doio} = B_{rodoio}+B_{radoio}+B_{rbdoio}$
$I_{doio} = I_{rodoio}+I_{radoio}+I_{rbdoio}$

init $L_{rodoio} = L_{rodoio1}$
init $L_{radoio} = 0$
init $L_{rbdoio} = 0$
init $B_{rodoio} = B_{rodoio1}$
init $B_{radoio} = 0$
init $B_{rbdoio} = 0$
init $I_{rodoio} = p_o*I_{doio0}$
init $I_{radoio} = p_a*I_{doio0}$
init $I_{rbdoio} = p_b*I_{doio0}$

;$S_{doio}$
; Example of code for stochasticity $S_{doio}(t+dt) = max(poisson(S_{doio}+(\mu*N_0 - \mu*S_{doio} - \beta*S_{doio}*T_{Iro}/N_0 + \delta*I_{rodoio} + c_{Brodo}*B_{rodoio} + c_{Irodo}*I_{rodoio} - \beta*S_{doio}*T_{Ira}/N_0 + \delta*I_{radoio} + c_{Brado}*B_{radoio} + c_{Irado}*I_{radoio} - \beta*S_{doio}*T_{Irb}/N_0 + \delta*I_{rbdoio} + c_{Brbdo}*B_{rbdoio} + c_{Irbdo}*I_{rbdoio} + (1/X_{ao})*S_{daio} - \tau_1*S_{doio} + (1/(X_b-X_{ai}))*S_{dbio})*dt),0)$
; this form of code was substituted for every differential equation in the model to achieve stochasticity

$d/dt(S_{doio}) = \mu*N_0 - \mu*S_{doio} - \beta*S_{doio}*T_{Iro}/N_0 + \delta*I_{rodoio} + c_{Brodo}*B_{rodoio} + c_{Irodo}*I_{rodoio} - \beta*S_{doio}*T_{Ira}/N_0 + \delta*I_{radoio} + c_{Brado}*B_{radoio} + c_{Irado}*I_{radoio} - \beta*S_{doio}*T_{Irb}/N_0 + \delta*I_{rbdoio} + c_{Brbdo}*B_{rbdoio} + c_{Irbdo}*I_{rbdoio} + (1/X_{ao})*S_{daio} - \tau 1*S_{doio} + (1/(X_b-X_{ai}))*S_{dbio}$

;rodoio
$d/dt(L_{rodoio}) = \beta*S_{doio}*T_{Iro}/N_0 - (\mu+\gamma)*L_{rodoio} + (1/Xao)*L_{rodaio} - \tau_1*L_{rodoio} + (1/(X_b-X_{ai}))*L_{rodbio}$
$d/dt(B_{rodoio}) = \gamma*L_{rodoio} - (\mu+\sigma)*B_{rodoio} - c_{Brodo}*B_{rodoio} - \tau*B_{rodoio} + (1/X_{ao})*B_{rodaio} - \tau_1*B_{rodoio} - \tau_2*B_{rodoio} - \tau_{ab}*B_{rodoio} + (1/(X_b-X_{ai}))*B_{rodbio}$
$d/dt(I_{rodoio}) = \sigma*B_{rodoio} - (\mu+\delta)*I_{rodoio} - c_{Irodo}*I_{rodoio} - \tau*I_{rodoio} + (1/X_{ao})*I_{rodaio} - \tau_1*I_{rodoio} - \tau_2*I_{rodoio} - \tau_{ab}*I_{rodoio} + (1/(X_b-X_{ai}))*I_{rodbio}$

;radoio
$d/dt(L_{radoio}) = \beta*S_{doio}*T_{Ira}/N_0 - (\mu+\gamma)*L_{radoio} + (1/X_{ao})*L_{radaio} - \tau_1*L_{radoio} + (1/(X_b-X_{ai}))*L_{radbio}$
$d/dt(B_{radoio}) = \gamma*L_{radoio} - (\mu+\sigma)*B_{radoio} - c_{Brado}*B_{radoio} - \tau*B_{radoio} + (1/X_{ao})*B_{radaio} - \tau_1*B_{radoio} - \tau_2*B_{radoio} - \tau_{ab}*B_{radoio} + (1/(X_b-X_{ai}))*B_{radbio}$
$d/dt(I_{radoio}) = \sigma*B_{radoio} - (\mu+\delta)*I_{radoio} - c_{Irado}*I_{radoio} - \tau*I_{radoio} + (1/X_{ao})*I_{radaio} - \tau_1*I_{radoio} - \tau_2*I_{radoio} - \tau_{ab}*I_{radoio} + (1/(X_b-X_{ai}))*I_{radbio} + SQUAREPULSE(st,1/365)*mst$

;rbdoio
$d/dt(L_{rbdoio}) = \beta*S_{doio}*T_{Irb}/N_0 - (\mu+\gamma)*L_{rbdoio} + (1/X_{ao})*L_{rbdaio} - \tau_1*L_{rbdoio} + (1/(X_b-X_{ai}))*L_{rbdbio}$

$d/dt(B_{rbdoio}) = \gamma*L_{rbdoio} - (\mu+\sigma)*B_{rbdoio} - c_{Brbdo}*B_{rbdoio} - \tau*B_{rbdoio} + (1/X_{ao})*B_{rbdaio} - \tau_1*B_{rbdoio} - \tau_2*B_{rbdoio} - \tau_{ab}*B_{rbdoio} + (1/(X_b-X_{ai}))*B_{rbdbio}$

$d/dt(I_{rbdoio}) = \sigma*B_{rbdoio} - (\mu+\delta)*I_{rbdoio} - c_{Irbdo}*I_{rbdoio} - \tau*I_{rbdoio} + (1/X_{ao})*I_{rbdaio} - \tau_1*I_{rbdoio} - \tau_2*I_{rbdoio} - \tau_{ab}*I_{rbdoio} + (1/(X_b-X_{ai}))*I_{rbdbio}$

;Box $N_{daio}$

init $S_{daio} = 0$

;init $B_{daio} = B_{daio0}$

;init $L_{daio} = L_{daio0}$

;init $I_{daio} = I_{daio0}$

$L_{daio} = L_{rodaio}+L_{radaio}+L_{rbdaio}$

$B_{daio} = B_{rodaio}+B_{radaio}+B_{rbdaio}$

$I_{daio} = I_{rodaio}+I_{radaio}+I_{rbdaio}$

init $L_{rodaio} = 0$

init $L_{radaio} = 0$

init $L_{rbdaio} = 0$

init $B_{rodaio} = 0$

init $B_{radaio} = 0$

init $B_{rbdaio} = 0$

init $I_{rodaio} = 0$

init $I_{radaio} = 0$

init $I_{rbdaio} = 0$

$d/dt(S_{daio}) = - \mu*S_{daio} - \beta*S_{daio}*T_{Iro}/N_0 + \delta*I_{rodaio} + c_{Broda}*B_{rodaio} + c_{Iroda}*I_{rodaio} - \beta*S_{daio}*T_{Ira}/N_0 + \delta*I_{radaio} + c_{Brada}*B_{radaio} + c_{Irada}*I_{radaio} - \beta*S_{daio}*T_{Irb}/N_0 + \delta*I_{rbdaio} + c_{Brbda}*B_{rbdaio} + c_{Irbda}*I_{rbdaio} - (1/X_{ao})*S_{daio} - \tau_1*S_{daio}$

;rodaio

$d/dt(L_{rodaio}) = \beta*S_{daio}*T_{Iro}/N_0 - (\mu+\gamma)*L_{rodaio} - (1/X_{ao})*L_{rodaio} - \tau 1*L_{rodaio}$

$d/dt(B_{rodaio}) = \gamma*L_{rodaio} - (\mu+\sigma)*B_{rodaio} - c_{Broda}*B_{rodaio} + \tau*B_{rodoio} - (1/X_{ao})*B_{rodaio} - \tau_1*B_{rodaio} - \tau_2*B_{rodaio}$

$d/dt(I_{rodaio}) = \sigma*B_{rodaio} - (\mu+\delta)*I_{rodaio} - c_{Iroda}*I_{rodaio} + \tau*I_{rodoio} - (1/X_{ao})*I_{rodaio} - \tau_1*I_{rodaio} - \tau_2*I_{rodaio}$

;radaio

$d/dt(L_{radaio}) = \beta*S_{daio}*T_{Ira}/N_0 - (\mu+\gamma)*L_{radaio} - (1/X_{ao})*L_{radaio} - \tau_1*L_{radaio}$

$d/dt(B_{radaio}) = \gamma*L_{radaio} - (\mu+\sigma)*B_{radaio} - c_{Brada}*B_{radaio} + \tau*B_{radoio} - (1/X_{ao})*B_{radaio} - \tau_1*B_{radaio} - \tau_2*B_{radaio}$

$d/dt(I_{radaio}) = \sigma*B_{radaio} - (\mu+\delta)*I_{radaio} - c_{Irada}*I_{radaio} + \tau*I_{radoio} - (1/X_{ao})*I_{radaio} - \tau_1*I_{radaio} - \tau_2*I_{radaio}$

;rbdaio

$d/dt(L_{rbdaio}) = \beta*S_{daio}*T_{Irb}/N_0 - (\mu+\gamma)*L_{rbdaio} - (1/X_{ao})*L_{rbdaio} - \tau 1*L_{rbdaio}$
$d/dt(B_{rbdaio}) = \gamma*L_{rbdaio} - (\mu+\sigma)*B_{rbdaio} - c_{Brbda}*B_{rbdaio} + \tau*B_{rbdoio} - (1/X_{ao})*B_{rbdaio} - \tau_1*B_{rbdaio} - \tau_2*B_{rbdaio}$
$d/dt(I_{rbdaio}) = \sigma*B_{rbdaio} - (\mu+\delta)*I_{rbdaio} - c_{Irbda}*I_{rbdaio} + \tau*I_{rbdoio} - (1/X_{ao})*I_{rbdaio} - \tau_1*I_{rbdaio} - \tau_2*I_{rbdaio}$

$N_{doio} = S_{doio} + L_{rodoio} + B_{rodoio} + I_{rodoio} + L_{radoio} + B_{radoio} + I_{radoio} + L_{rbdoio} + B_{rbdoio} + I_{rbdoio}$
$N_{daio} = S_{daio} + L_{rodaio} + B_{rodaio} + I_{rodaio} + L_{radaio} + B_{radaio} + I_{radaio} + L_{rbdaio} + B_{rbdaio} + I_{rbdaio}$

;Intervention 1
;MDA using artesunate/piperaquine +/- primaquine
;$N_{dabi1}$, $N_{dbi1}$, $N_{doi1}$

;Box $N_{dabi1}$

init $S_{dabi1} = 0$
;init $B_{dabi1} = B_{dabi1}0$
;init $L_{dabi1} = L_{dabi1}0$
;init $I_{dabi1} = I_{dabi1}0$

$L_{dabi1} = L_{rodabi1}+L_{radabi1}+L_{rbdabi1}$
$B_{dabi1} = B_{rodabi1}+B_{radabi1}+B_{rbdabi1}$
$I_{dabi1} = I_{rodabi1}+I_{radabi1}+I_{rbdabi1}$

init $L_{rodabi1} = 0$
init $L_{radabi1} = 0$
init $L_{rbdabi1} = 0$
init $B_{rodabi1} = 0$
init $B_{radabi1} = 0$
init $B_{rbdabi1} = 0$
init $I_{rodabi1} = 0$
init $I_{radabi1} = 0$
init $I_{rbdabi1} = 0$

$d/dt(S_{dabi1}) = - \mu*S_{dabi1} - \beta*S_{dabi1}*T_{Iro}/N_0 + \delta*I_{rodabi1} + c_{Brodab}*B_{rodabi1} + c_{Irodab}*I_{rodabi1} - \beta*S_{dabi1}*T_{Ira}/N_0 + \delta*I_{radabi1} + c_{Bradab}*B_{radabi1} + c_{Iradab}*I_{radabi1} - \beta*S_{dabi1}*T_{Irb}/N_0 + \delta*I_{rbdabi1} + c_{Brbdab}*B_{rbdabi1} + c_{Irbdab}*I_{rbdabi1} + \tau_1*S_{doio} + \tau_1*S_{daio} - (1/X_{ai})*S_{dabi1} + c_{Lp}*L_{rodabi1} + c_{Ip}*I_{rodabi1} + c_{Lp}*L_{radabi1} + c_{Ip}*I_{radabi1} + c_{Lp}*L_{rbdabi1} + c_{Ip}*I_{rbdabi1}$

;rodabi1

$d/dt(L_{rodabi1}) = \beta*S_{dabi1}*T_{Iro}/N_0 - (\mu+\gamma)*L_{rodabi1} + \tau_1*L_{rodoio} + \tau_1*L_{rodaio} - (1/X_{ai})*L_{rodabi1} - c_{Lp}*L_{rodabi1}$
$d/dt(B_{rodabi1}) = \gamma*L_{rodabi1} - (\mu+\sigma)*B_{rodabi1} - c_{Brodab}*B_{rodabi1} + \tau_1*B_{rodoio} + \tau_1*B_{rodaio} - (1/X_{ai})*B_{rodabi1} + \tau_{abi1}*B_{rodoi1} + \tau_1*B_{rodoi1}$
$d/dt(I_{rodabi1}) = \sigma*B_{rodabi1} - (\mu+\delta)*I_{rodabi1} - c_{Irodab}*I_{rodabi1} + \tau_1*I_{rodoio} + \tau_1*I_{rodaio} - (1/X_{ai})*I_{rodabi1} + \tau_{abi1}*I_{rodoi1} - c_{Ip}*I_{rodabi1} + \tau_1*I_{rodoi1}$

;radabi1
$d/dt(L_{radabi1}) = \beta*S_{dabi1}*T_{Ira}/N_0 - (\mu+\gamma)*L_{radabi1} + \tau_1*L_{radoio} +\tau_1*L_{radaio} - (1/X_{ai})*L_{radabi1} - c_{Lp}*L_{radabi1}$
$d/dt(B_{radabi1}) = \gamma*L_{radabi1} - (\mu+\sigma)*B_{radabi1} - c_{Bradab}*B_{radabi1} + \tau_1*B_{radoio} + \tau_1*B_{radaio} - (1/X_{ai})*B_{radabi1} + \tau_{abi1}*B_{radoi1} + \tau_1*B_{radoi1}$
$d/dt(I_{radabi1}) = \sigma*B_{radabi1} - (\mu+\delta)*I_{radabi1} - c_{Iradab}*I_{radabi1} + \tau_1*I_{radoio} + \tau_1*I_{radaio} - (1/X_{ai})*I_{radabi1} + \tau_{abi1}*I_{radoi1} - c_{Ip}*I_{radabi1} + \tau_1*I_{radoi1}$

;rbdabi1
$d/dt(L_{rbdabi1}) = \beta*S_{dabi1}*T_{Irb}/N_0 - (\mu+\gamma)*L_{rbdabi1} + \tau_1*L_{rbdoio} +\tau_1*L_{rbdaio} - (1/X_{ai})*L_{rbdabi1} - c_{Lp}*L_{rbdabi1}$
$d/dt(B_{rbdabi1}) = \gamma*L_{rbdabi1} - (\mu+\sigma)*B_{rbdabi1} - c_{Brbdab}*B_{rbdabi1} + \tau_1*B_{rbdoio} + \tau_1*B_{rbdaio} - (1/X_{ai})*B_{rbdabi1} + \tau_{abi1}*B_{rbdoi1} + \tau_{abi1}*B_{rbdbi1} + \tau_1*B_{rbdoi1}$
$d/dt(I_{rbdabi1}) = \sigma*B_{rbdabi1} - (\mu+\delta)*I_{rbdabi1} - c_{Irbdab}*I_{rbdabi1} + \tau_1*I_{rbdoio} + \tau_1*I_{rbdaio} - (1/X_{ai})*I_{rbdabi1} + \tau_{abi1}*I_{rbdoi1}+ \tau_{abi1}*I_{rbdbi1} - c_{Ip}*I_{rbdabi1} + \tau_1*I_{rbdoi1}$

$N_{dabi1} = S_{dabi1} + L_{rodabi1} + B_{rodabi1} + I_{rodabi1} + L_{radabi1} + B_{radabi1} + I_{radabi1} + L_{rbdabi1} + B_{rbdabi1} + I_{rbdabi1}$

;Box $N_{dbi1}$

init $S_{dbi1} = 0$
;init $B_{dbi1} = B_{dbi1}0$
;init $L_{dbi1} = L_{dbi1}0$
;init $I_{dbi1} = I_{dbi1}0$

$L_{dbi1} = L_{rodbi1}+L_{radbi1}+L_{rbdbi1}$
$B_{dbi1} = B_{rodbi1}+B_{radbi1}+B_{rbdbi1}$
$I_{dbi1} = I_{rodbi1}+I_{radbi1}+I_{rbdbi1}$

init $L_{rodbi1} = 0$
init $L_{radbi1} = 0$
init $L_{rbdbi1} = 0$
init $B_{rodbi1} = 0$
init $B_{radbi1} = 0$

init $B_{rbdbi1} = 0$
init $I_{rodbi1} = 0$
init $I_{radbi1} = 0$
init $I_{rbdbi1} = 0$

$d/dt(S_{dbi1}) = - \mu*S_{dbi1} - \beta*S_{dbi1}*TIro/N_0 + \delta*I_{rodbi1} + c_{Brodb}*B_{rodbi1} + c_{Irodb}*I_{rodbi1} - \beta*S_{dbi1}*T_{Ira}/N_0 + \delta*I_{radbi1} + c_{Bradb}*B_{radbi1} + c_{Iradb}*I_{radbi1} - \beta*S_{dbi1}*T_{Irb}/N_0 + \delta*I_{rbdbi1} + c_{Brbdb}*B_{rbdbi1} + c_{Irbdb}*I_{rbdbi1} + (1/X_{ai})*S_{dabi1} - (1/(X_b-X_{ai})*S_{dbi1})$

;rodbi1
$d/dt(L_{rodbi1}) = \beta*S_{dbi1}*T_{Iro}/N_0 - (\mu+\gamma)*L_{rodbi1} + (1/X_{ai})*L_{rodabi1} - (1/(X_b-X_{ai})*L_{rodbi1})$
$d/dt(B_{rodbi1}) = \gamma*L_{rodbi1} - (\mu+\sigma)*B_{rodbi1} - c_{Brodb}*B_{rodbi1} + (1/X_{ai})*B_{rodabi1} - (1/(X_b-X_{ai})*B_{rodbi1})$
$d/dt(I_{rodbi1}) = \sigma*B_{rodbi1} - (\mu+\delta)*I_{rodbi1} - c_{Irodb}*I_{rodbi1} + (1/X_{ai})*I_{rodabi1} - (1/(X_b-X_{ai})*I_{rodbi1})$

;radbi1
$d/dt(L_{radbi1}) = \beta*S_{dbi1}*T_{Ira}/N_0 - (\mu+\gamma)*L_{radbi1} + (1/X_{ai})*L_{radabi1} - (1/(X_b-X_{ai})*L_{radbi1})$
$d/dt(B_{radbi1}) = \gamma*L_{radbi1} - (\mu+\sigma)*B_{radbi1} - c_{Bradb}*B_{radbi1} + (1/X_{ai})*B_{radabi1} - (1/(X_b-X_{ai})*B_{radbi1})$
$d/dt(I_{radbi1}) = \sigma*B_{radbi1} - (\mu+\delta)*I_{radbi1} - c_{\underline{Iradb}}*I_{radbi1} + (1/X_{ai})*I_{radabi1} - (1/(X_b-X_{ai})*I_{radbi1})$

;rbdbi1
$d/dt(L_{rbdbi1}) = \beta*S_{dbi1}*T_{Irb}/N_0 - (\mu+\gamma)*L_{rbdbi1} + (1/X_{ai})*L_{rbdabi1} - (1/(X_b-X_{ai})*L_{rbdbi1})$
$d/dt(B_{rbdbi1}) = \gamma*L_{rbdbi1} - (\mu+\sigma)*B_{rbdbi1} - c_{Brbdb}*B_{rbdbi1} + (1/X_{ai})*B_{rbdabi1} - (1/(X_b-X_{ai})*B_{rbdbi1}) - \tau abi1*B_{rbdbi1} - \tau_{ai}1*B_{rbdbi1}$
$d/dt(I_{rbdbi1}) = \sigma*B_{rbdbi1} - (\mu+\delta)*I_{rbdbi1} - c_{Irbdb}*I_{rbdbi1} + (1/X_{ai})*I_{rbdabi1} - (1/(X_b-X_{ai})*I_{rbdbi1}) - \tau_{abi1}*I_{rbdbi1} - \tau ai1*I_{rbdbi1}$

$N_{dbi1} = S_{dbi1} + L_{rodbi1} + B_{rodbi1} + I_{rodbi1} + L_{radbi1} + B_{radbi1} + I_{radbi1} + L_{rbdbi1} + B_{rbdbi1} + I_{rbdbi1}$

;Box $N_{doi1}$

init $S_{doi1} = 0;N_0-I_{doi1}0$
;init $B_{doi1} = B_{doi1}0$
;init $L_{doi1} = L_{doi1}0$
$I_{doi1}0 = 0;N_0*p_{inf}$

$L_{doi1} = L_{rodoi1}+L_{radoi1}+L_{rbdoi1}$
$B_{doi1} = B_{rodoi1}+B_{radoi1}+B_{rbdoi1}$
$I_{doi1} = I_{rodoi1}+I_{radoi1}+I_{rbdoi1}$

init $L_{rodoi1} = 0$

init $L_{radoi1} = 0$
init $L_{rbdoi1} = 0$
init $B_{rodoi1} = 0$
init $B_{radoi1} = 0$
init $B_{rbdoi1} = 0$
init $I_{rodoi1} = 0;p_o*I_{doi1}0$
init $I_{radoi1} = 0;p_a*I_{doi1}0$
init $I_{rbdoi1} = 0;p_b*I_{doi1}0$

$d/dt(S_{doi1}) = - \mu*S_{doi1} - \beta*S_{doi1}*T_{Iro}/N_0 + \delta*I_{rodoi1} + c_{Brodo}*B_{rodoi1} + c_{Irodo}*I_{rodoi1} - \beta*S_{doi1}*T_{Ira}/N_0 + \delta*I_{radoi1} + c_{Brado}*B_{radoi1} + c_{Irado}*I_{radoi1} - \beta*S_{doi1}*T_{Irb}/N_0 + \delta*I_{rbdoi1} + c_{Brbdo}*B_{rbdoi1} + c_{Irbdo}*I_{rbdoi1} + (1/(X_b-X_{ai}))*S_{dbi1} + 1/(X_b-X_{ai}-X_{ao})*S_{dbi1a} + (1/X_{ao})*S_{dai1a}$

;rodoi1
$d/dt(L_{rodoi1}) = \beta*S_{doi1}*T_{Iro}/N_0 - (\mu+\gamma)*L_{rodoi1} + (1/(X_b-X_{ai}))*L_{rodbi1} + 1/(X_b-X_{ai}-X_{ao})*L_{rodbi1a} + (1/X_{ao})*L_{rodai1a}$
$d/dt(B_{rodoi1}) = \gamma*L_{rodoi1} - (\mu+\sigma)*B_{rodoi1} - c_{Brodo}*B_{rodoi1} + (1/(X_b-X_{ai}))*B_{rodbi1} - \tau_{abi1}*B_{rodoi1} + 1/(X_b-X_{ai}-X_{ao})*B_{rodbi1a} - \tau_{ai1}*B_{rodoi1} + (1/X_{ao})*B_{rodai1a} - \tau_1*B_{rodoi1}$
$d/dt(I_{rodoi1}) = \sigma*B_{rodoi1} - (\mu+\delta)*I_{rodoi1} - c_{Irodo}*I_{rodoi1} + (1/(X_b-X_{ai}))*I_{rodbi1} - \tau_{abi1}*I_{rodoi1} + 1/(X_b-X_{ai}-X_{ao})*I_{rodbi1a} - \tau_{ai1}*I_{rodoi1} + (1/X_{ao})*I_{rodai1a} - \tau_1*I_{rodoi1}$

;radoi1
$d/dt(L_{radoi1}) = \beta*S_{doi1}*T_{Ira}/N_0 - (\mu+\gamma)*L_{radoi1} + (1/(X_b-X_{ai}))*L_{radbi1} + 1/(X_b-X_{ai}-X_{ao})*L_{radbi1a} + (1/X_{ao})*L_{radai1a}$
$d/dt(B_{radoi1}) = \gamma*L_{radoi1} - (\mu+\sigma)*B_{radoi1} - c_{Brado}*B_{radoi1} + (1/(X_b-X_{ai}))*B_{radbi1} - \tau_{abi1}*B_{radoi1} + 1/(X_b-X_{ai}-X_{ao})*B_{radbi1a} - \tau_{ai1}*B_{radoi1} + (1/X_{ao})*B_{radai1a} - \tau 1*B_{radoi1}$
$d/dt(I_{radoi1}) = \sigma*B_{radoi1} - (\mu+\delta)*I_{radoi1} - c_{Irado}*I_{radoi1} + (1/(X_b-X_{ai}))*I_{radbi1} - \tau_{abi1}*I_{radoi1} + 1/(X_b-X_{ai}-X_{ao})*I_{radbi1a} - \tau_{ai1}*I_{radoi1} + (1/X_{ao})*I_{radai1a} - \tau_1*I_{radoi1}$

;rbdoi1
$d/dt(L_{rbdoi1}) = \beta*S_{doi1}*T_{Irb}/N_0 - (\mu+\gamma)*L_{rbdoi1} + (1/(X_b-X_{ai}))*L_{rbdbi1} + 1/(X_b-X_{ai}-X_{ao})*L_{rbdbi1a} + (1/X_{ao})*L_{rbdai1a}$
$d/dt(B_{rbdoi1}) = \gamma*L_{rbdoi1} - (\mu+\sigma)*B_{rbdoi1} - c_{Brbdo}*B_{rbdoi1} + (1/(X_b-X_{ai}))*B_{rbdbi1} - \tau_{abi1}*B_{rbdoi1} + 1/(X_b-X_{ai}-X_{ao})*B_{rbdbi1a} - \tau_{ai1}*B_{rbdoi1} + (1/X_{ao})*B_{rbdai1a} - \tau_1*B_{rbdoi1}$
$d/dt(I_{rbdoi1}) = \sigma*B_{rbdoi1} - (\mu+\delta)*I_{rbdoi1} - c_{Irbdo}*I_{rbdoi1} + (1/(X_b-X_{ai}))*I_{rbdbi1} - \tau_{abi1}*I_{rbdoi1} + 1/(X_b-X_{ai}-X_{ao})*I_{rbdbi1a} - \tau_{ai1}*I_{rbdoi1} + (1/X_{ao})*I_{rbdai1a} - \tau_1*I_{rbdoi1}$

$N_{doi1} = S_{doi1} + L_{rodoi1} + B_{rodoi1} + {}_{Irodoi1} + L_{radoi1} + B_{radoi1} + {}_{Iradoi1} + L_{rbdoi1} + B_{rbdoi1} + I_{rbdoi1}$

;Treatment with artesunate in Intervention 1
; $N_{dai1a}$

;Box $N_{dai1a}$

init $S_{dai1a} = 0$
;init $B_{dai1a} = B_{dai1a}0$
;init $L_{dai1a} = L_{dai1a}0$
;init $I_{dai1a} = I_{dai1a}0$

$L_{dai1a} = L_{rodai1a}+L_{radai1a}+L_{rbdai1a}$
$B_{dai1a} = B_{rodai1a}+B_{radai1a}+B_{rbdai1a}$
$I_{dai1a} = I_{rodai1a}+I_{radai1a}+I_{rbdai1a}$

init $L_{rodai1a} = 0$
init $L_{radai1a} = 0$
init $L_{rbdai1a} = 0$
init $B_{rodai1a} = 0$
init $B_{radai1a} = 0$
init $B_{rbdai1a} = 0$
init $I_{rodai1a} = 0$
init $I_{radai1a} = 0$
init $I_{rbdai1a} = 0$

$d/dt(S_{dai1a}) = - \mu*S_{dai1a} - \beta*S_{dai1a}*T_{Iro}/N_0 + \delta*I_{rodai1a} + c_{Broda}*B_{rodai1a} + c_{Iroda}*I_{rodai1a} - \beta*S_{dai1a}*T_{Ira}/N_0 + \delta*I_{radai1a} + c_{Brada}*B_{radai1a} + c_{Irada}*I_{radai1a} - \beta*S_{dai1a}*T_{Irb}/N_0 + \delta*I_{rbdai1a} + c_{Brbda}*B_{rbdai1a} + c_{Irbda}*I_{rbdai1a} - (1/X_{ao})*S_{dai1a}$

;rodai1a
$d/dt(L_{rodai1a}) = \beta*S_{dai1a}*T_{Iro}/N_0 - (\mu+\gamma)*L_{rodai1a} - (1/X_{ao})*L_{rodai1a}$
$d/dt(B_{rodai1a}) = \gamma*L_{rodai1a} - (\mu+\sigma)*B_{rodai1a} - c_{Broda}*B_{rodai1a} + \tau_{ai1}*B_{rodoi1} - (1/X_{ao})*B_{rodai1a}$
$d/dt(I_{rodai1a}) = \sigma*B_{rodai1a} - (\mu+\delta)*I_{rodai1a} - c_{Iroda}*I_{rodai1a} + \tau_{ai1}*I_{rodoi1} - (1/X_{ao})*I_{rodai1a}$

;radai1a
$d/dt(L_{radai1a}) = \beta*S_{dai1a}*T_{Ira}/N_0 - (\mu+\gamma)*L_{radai1a} - (1/X_{ao})*L_{radai1a}$
$d/dt(B_{radai1a}) = \gamma*L_{radai1a} - (\mu+\sigma)*B_{radai1a} - c_{Brada}*B_{radai1a} + \tau_{ai1}*B_{radoi1} - (1/X_{ao})*B_{radai1a}$
$d/dt(I_{radai1a}) = \sigma*B_{radai1a} - (\mu+\delta)*I_{radai1a} - c_{Irada}*I_{radai1a} + \tau_{ai1}*I_{radoi1} - (1/X_{ao})*I_{radai1a}$

;rbdai1a
$d/dt(L_{rbdai1a}) = \beta*S_{dai1a}*T_{Irb}/N_0 - (\mu+\gamma)*L_{rbdai1a} - (1/X_{ao})*L_{rbdai1a}$
$d/dt(B_{rbdai1a}) = \gamma*L_{rbdai1a} - (\mu+\sigma)*B_{rbdai1a} - c_{Brbda}*B_{rbdai1a} + \tau_{ai1}*B_{rbdoi1} - (1/X_{ao})*B_{rbdai1a}$
$d/dt(I_{rbdai1a}) = \sigma*B_{rbdai1a} - (\mu+\delta)*I_{rbdai1a} - c_{Irbda}*I_{rbdai1a} + \tau_{ai1}*I_{rbdoi1} - (1/X_{ao})*I_{rbdai1a}$

$N_{dai1a} = S_{dai1a} + L_{rodai1a} + B_{rodai1a} + I_{rodai1a} + L_{radai1a} + B_{radai1a} + I_{radai1a} + L_{rbdai1a} + B_{rbdai1a} + I_{rbdai1a}$

;Treatment of infected patients with artesunate/piperaquine in Intervention 1
; $N_{dabi1a,}$ $N_{dbi1a}$

;Box $N_{dabi1a}$

init $S_{dabi1a} = 0$
;init $B_{dabi1a} = B_{dabi1a}0$
;init $L_{dabi1a} = L_{dabi1a}0$
;init $I_{dabi1a} = I_{dabi1a}0$

$L_{dabi1a} = L_{rodabi1a}+L_{radabi1a}+L_{rbdabi1a}$
$B_{dabi1a} = B_{rodabi1a}+B_{radabi1a}+B_{rbdabi1a}$
$I_{dabi1a} = {}_{Irodabi1a}+{}_{Iradabi1a}+I_{rbdabi1a}$

init $L_{rodabi1a} = 0$
init $L_{radabi1a} = 0$
init $L_{rbdabi1a} = 0$
init $B_{rodabi1a} = 0$
init $B_{radabi1a} = 0$
init $B_{rbdabi1a} = 0$
init ${}_{Irodabi1a} = 0$
init ${}_{Iradabi1a} = 0$
init $I_{rbdabi1a} = 0$

$d/dt(S_{dabi1a}) = - \mu*S_{dabi1a} - \beta*S_{dabi1a}*T_{Iro}/N_0 + \delta*_{Irodabi1a} + c_{Brodab}*B_{rodabi1a} + c_{Irodab}*_{Irodabi1a} - \beta*S_{dabi1a}*T_{Ira}/N_0 + \delta*_{Iradabi1a} + c_{Bradb}*B_{radabi1a} + c_{Iradab}*_{Iradabi1a} - \beta*S_{dabi1a}*T_{Irb}/N_0 + \delta*I_{rbdabi1a} + c_{Brbdab}*B_{rbdabi1a} + c_{Irbdab}*I_{rbdabi1a} - (1/X_{ao}*S_{dabi1a})$

;rodabi1a
$d/dt(L_{rodabi1a}) = \beta*S_{dabi1a}*T_{Iro}/N_0 - (\mu+\gamma)*L_{rodabi1a} - (1/X_{ao})*L_{rodabi1a}$
$d/dt(B_{rodabi1a}) = \gamma*L_{rodabi1a} - (\mu+\sigma)*B_{rodabi1a} - c_{Brodab}*B_{rodabi1a} - (1/X_{ao})*B_{rodabi1a}$
$d/dt(_{Irodabi1a}) = \sigma*B_{rodabi1a} - (\mu+\delta)*_{Irodabi1a} - c_{Irodab}*_{Irodabi1a} - (1/X_{ao})*_{Irodabi1a}$

;radabi1a
$d/dt(L_{radabi1a}) = \beta*S_{dabi1a}*T_{Ira}/N_0 - (\mu+\gamma)*L_{radabi1a} - (1/X_{ao})*L_{radabi1a}$
$d/dt(B_{radabi1a}) = \gamma*L_{radabi1a} - (\mu+\sigma)*B_{radabi1a} - c_{Bradab}*B_{radabi1a} - (1/X_{ao})*B_{radabi1a}$
$d/dt(_{Iradabi1a}) = \sigma*B_{radabi1a} - (\mu+\delta)*_{Iradabi1a} - c_{Iradab}*_{Iradabi1a} - (1/X_{ao})*_{Iradabi1a}$

;rbdabi1a
$d/dt(L_{rbdabi1a}) = \beta*S_{dabi1a}*TI_{rb}/N_0 - (\mu+\gamma)*L_{rbdabi1a} - (1/X_{ao})*L_{rbdabi1a}$

$d/dt(B_{rbdabi1a}) = \gamma*L_{rbdabi1a} - (\mu+\sigma)*B_{rbdabi1a} - c_{Brbdab}*B_{rbdabi1a} + \tau_{ai1}*B_{rbdbi1a} - (1/X_{ao})*B_{rbdabi1a} + \tau_{ai1}*B_{rbdbi1}$
$d/dt(I_{rbdabi1a}) = \sigma*B_{rbdabi1a} - (\mu+\delta)*I_{rbdabi1a} - c_{Irbdab}*I_{rbdabi1a} + \tau_{ai1}*I_{rbdbi1a} - (1/X_{ao})*I_{rbdabi1a} + \tau_{ai1}*I_{rbdbi1}$

$N_{dabi1a} = S_{dabi1a} + L_{rodabi1a} + B_{rodabi1a} + I_{rodabi1a} + L_{radabi1a} + B_{radabi1a} + I_{radabi1a} + L_{rbdabi1a} + B_{rbdabi1a} + I_{rbdabi1a}$

;Box $N_{dbi1a}$

init $S_{dbi1a} = 0$
;init $B_{dbi1a} = B_{dbi1a}0$
;init $L_{dbi1a} = L_{dbi1a}0$
;init $I_{dbi1a} = I_{dbi1a}0$

$L_{dbi1a} = L_{rodbi1a}+L_{radbi1a}+L_{rbdbi1a}$
$B_{dbi1a} = B_{rodbi1a}+B_{radbi1a}+B_{rbdbi1a}$
$I_{dbi1a} = I_{rodbi1a}+I_{radbi1a}+I_{rbdbi1a}$

init $L_{rodbi1a} = 0$
init $L_{radbi1a} = 0$
init $L_{rbdbi1a} = 0$
init $B_{rodbi1a} = 0$
init $B_{radbi1a} = 0$
init $B_{rbdbi1a} = 0$
init $I_{rodbi1a} = 0$
init $I_{radbi1a} = 0$
init $I_{rbdbi1a} = 0$

$d/dt(S_{dbi1a}) = - \mu*S_{dbi1a} - \beta*S_{dbi1a}*T_{Iro}/N_0 + \delta*I_{rodbi1a} + c_{Brodb}*B_{rodbi1a} + c_{Irodb}*I_{rodbi1a} - \beta*S_{dbi1a}*T_{Ira}/N_0 + \delta*I_{radbi1a} + c_{Bradb}*B_{radbi1a} + c_{Iradb}*I_{radbi1a} - \beta*S_{dbi1a}*T_{Irb}/N_0 + \delta*I_{rbdbi1a} + c_{Brbdb}*B_{rbdbi1a} + c_{Irbdb}*I_{rbdbi1a} + (1/X_{ao}*S_{dabi1a}) - 1/(X_b-X_{ai}-X_{ao})*S_{dbi1a}$

;rodbi1a
$d/dt(L_{rodbi1a}) = \beta*S_{dbi1a}*T_{Iro}/N_0 - (\mu+\gamma)*L_{rodbi1a} + (1/X_{ao})*L_{rodabi1a} - 1/(X_b-X_{ai}-X_{ao})*L_{rodbi1a}$
$d/dt(B_{rodbi1a}) = \gamma*L_{rodbi1a} - (\mu+\sigma)*B_{rodbi1a} - c_{Brodb}*B_{rodbi1a} + (1/X_{ao})*B_{rodabi1a} - 1/(X_b-X_{ai}-X_{ao})*B_{rodbi1a}$
$d/dt(I_{rodbi1a}) = \sigma*B_{rodbi1a} - (\mu+\delta)*I_{rodbi1a} - c_{Irodb}*I_{rodbi1a} + (1/X_{ao})*I_{rodabi1a} - 1/(X_b-X_{ai}-X_{ao})*I_{rodbi1a}$

;radbi1a
$d/dt(L_{radbi1a}) = \beta*S_{dbi1a}*T_{Ira}/N_0 - (\mu+\gamma)*L_{radbi1a} + (1/X_{ao})*L_{radabi1a} - 1/(X_b-X_{ai}-X_{ao})*L_{radbi1a}$
$d/dt(B_{radbi1a}) = \gamma*L_{radbi1a} - (\mu+\sigma)*B_{radbi1a} - c_{Bradb}*B_{radbi1a} + (1/X_{ao})*B_{radabi1a} - 1/(X_b-X_{ai}-X_{ao})*B_{radbi1a}$

$d/dt(I_{radbi1a}) = \sigma*B_{radbi1a} - (\mu+\delta)*I_{radbi1a} - c_{Ira}db*I_{radbi1a} + (1/X_{ao})*I_{radabi1a} - 1/(X_b-X_{ai}-X_{ao})*I_{radbi1a}$

;rbdbi1a
$d/dt(L_{rbdbi1a}) = \beta*S_{dbi1a}*T_{Irb}/N_0 - (\mu+\gamma)*L_{rbdbi1a} + (1/X_{ao})*L_{rbdabi1a} - 1/(X_b-X_{ai}-X_{ao})*L_{rbdbi1a}$
$d/dt(B_{rbdbi1a}) = \gamma*L_{rbdbi1a} - (\mu+\sigma)*B_{rbdbi1a} - c_{Brbdb}*B_{rbdbi1a} - \tau_{ai1}*B_{rbdbi1a} + (1/X_{ao})*B_{rbdabi1a} - 1/(X_b-X_{ai}-X_{ao})*B_{rbdbi1a}$
$d/dt(I_{rbdbi1a}) = \sigma*B_{rbdbi1a} - (\mu+\delta)*I_{rbdbi1a} - c_{Irbdb}*I_{rbdbi1a} - \tau_{ai1}*I_{rbdbi1a} + (1/X_{ao})*I_{rbdabi1a} - 1/(X_b-X_{ai}-X_{ao})*I_{rbdbi1a}$

$N_{dbi1a} = S_{dbi1a} + L_{rodbi1a} + B_{rodbi1a} + I_{rodbi1a} + L_{radbi1a} + B_{radbi1a} + I_{radbi1a} + L_{rbdbi1a} + B_{rbdbi1a} + I_{rbdbi1a}$

;Switch to ACT (artesunate + piperaquine) for treatment in place of artesunate monotherapy
;also Intervention 3: MSAT with ACT
; $N_{dabio,}$ $N_{dbio}$

;Box $N_{dabio}$

init $S_{dabio} = 0$
;init $B_{dabio} = B_{dabio}0$
;init $L_{dabio} = L_{dabio}0$
;init $I_{dabio} = I_{dabio}0$

$L_{dabio} = L_{rodabio}+L_{radabio}+L_{rbdabio}$
$B_{dabio} = B_{rodabio}+B_{radabio}+B_{rbdabio}$
$I_{dabio} = {}_{Irodabio}+{}_{Iradabio}+I_{rbdabio}$

init $L_{rodabio} = 0$
init $L_{radabio} = 0$
init $L_{rbdabio} = 0$
init $B_{rodabio} = 0$
init $B_{radabio} = 0$
init $B_{rbdabio} = 0$
init $I_{rodabio} = 0$
init $I_{radabio} = 0$
init $I_{rbdabio} = 0$

$d/dt(S_{dabio}) = - \mu*S_{dabio} - \beta*S_{dabio}*T_{Iro}/N_0 + \delta*I_{rodabio} + c_{Brodab}*B_{rodabio} + c_{Irodab}*I_{rodabio} - \beta*S_{dabio}*T_{Ira}/N_0 + \delta*I_{radabio} + c_{Bradab}*B_{radabio} + c_{Iradab}*I_{radabio} - \beta*S_{dabio}*T_{Irb}/N_0 + \delta*I_{rbdabio} + c_{Brbdab}*B_{rbdabio} + c_{Irbdab}*I_{rbdabio} - (1/X_{ai})*S_{dabio} + c_{Lp}*(L_{rodabio} + L_{radabio} + L_{rbdabio}) + c_{Ip}*(I_{rodabio} + I_{radabio} + I_{rbdabio})$

;rodabio
$d/dt(L_{rodabio}) = \beta*S_{dabio}*T_{Iro}/N_0 - (\mu+\gamma)*L_{rodabio} - (1/X_{ai})*L_{rodabio} - c_{Lp}*L_{rodabio}$
$d/dt(B_{rodabio}) = \gamma*L_{rodabio} - (\mu+\sigma)*B_{rodabio} - c_{Brodab}*B_{rodabio} + \tau_{ab}*B_{rodoio} - (1/X_{ai})*B_{rodabio}$
$d/dt(I_{rodabio}) = \sigma*B_{rodabio} - (\mu+\delta)*I_{rodabio} - c_{Irodab}*I_{rodabio} + \tau_{ab}*I_{rodoio} - (1/X_{ai})*I_{rodabio} -c_{Ip}*I_{rodabio}$

;radabio
$d/dt(L_{radabio}) = \beta*S_{dabio}*T_{Ira}/N_0 - (\mu+\gamma)*L_{radabio} - (1/X_{ai})*L_{radabio} - c_{Lp}*L_{radabio}$
$d/dt(B_{radabio}) = \gamma*L_{radabio} - (\mu+\sigma)*B_{radabio} - c_{Bradab}*B_{radabio} + \tau_{ab}*B_{radoio} - (1/X_{ai})*B_{radabio}$
$d/dt({}_{Iradabio}) = \sigma*B_{radabio} - (\mu+\delta)*{}_{Iradabio} - c_{Iradab}*{}_{Iradabio} + \tau ab*I_{radoio} - (1/X_{ai})*I_{radabio} - c_{Ip}*I_{radabio}$

;rbdabio
$d/dt(L_{rbdabio}) = \beta*S_{dabio}*T_{Irb}/N_0 - (\mu+\gamma)*L_{rbdabio} - (1/X_{ai})*L_{rbdabio} - c_{Lp}*L_{rbdabio}$
$d/dt(B_{rbdabio}) = \gamma*L_{rbdabio} - (\mu+\sigma)*B_{rbdabio} - c_{Brbdab}*B_{rbdabio} + \tau_{ab}*B_{rbdoio} - (1/X_{ai})*B_{rbdabio} + \tau_{ab}*B_{rbdbio}$
$d/dt(I_{rbdabio}) = \sigma*B_{rbdabio} - (\mu+\delta)*I_{rbdabio} - c_{Irbdab}*I_{rbdabio} + \tau_{ab}*I_{rbdoio} - (1/X_{ai})*I_{rbdabio} + \tau_{ab}*I_{rbdbio} - c_{Ip}*I_{rbdabio}$

$N_{dabio} = S_{dabio} + L_{rodabio} + B_{rodabio} + I_{rodabio} + L_{radabio} + B_{radabio} + I_{radabio} + L_{rbdabio} + B_{rbdabio} + I_{rbdabio}$

;Box $N_{dbio}$

init $S_{dbio} = 0$
;init $B_{dbio} = B_{dbio}0$
;init $L_{dbio} = L_{dbio}0$
;init $I_{dbio} = I_{dbio}0$

$L_{dbio} = L_{rodbio}+L_{radbio}+L_{rbdbio}$
$B_{dbio} = B_{rodbio}+B_{radbio}+B_{rbdbio}$
$I_{dbio} = I_{rodbio}+I_{radbio}+I_{rbdbio}$

init $L_{rodbio} = 0$
init $L_{radbio} = 0$
init $L_{rbdbio} = 0$
init $B_{rodbio} = 0$
init $B_{radbio} = 0$
init $B_{rbdbio} = 0$
init ${}_{Irodbio} = 0$
init ${}_{Iradbio} = 0$
init $I_{rbdbio} = 0$

$d/dt(S_{dbio}) = - \mu*S_{dbio} - \beta*S_{dbio}*T_{Iro}/N_0 + \delta*I_{rodbio} + c_{Brodb}*B_{rodbio} + c_{Irodb}*I_{rodbio} - \beta*S_{dbio}*T_{Ira}/N_0 + \delta*I_{radbio} + c_{Bradb}*B_{radbio} + c_{Iradb}*I_{radbio} - \beta*S_{dbio}*T_{Irb}/N_0 + \delta*I_{rbdbio} + c_{Brbdb}*B_{rbdbio} + c_{Irbdb}*I_{rbdbio} + (1/X_{ai})*S_{dabio} - (1/(X_b-X_{ai})*S_{dbio})$

;rodbio
$d/dt(L_{rodbio}) = \beta*S_{dbio}*T_{Iro}/N_0 - (\mu+\gamma)*L_{rodbio} + (1/X_{ai})*L_{rodabio} - (1/(X_b-X_{ai})*L_{rodbio})$
$d/dt(B_{rodbio}) = \gamma*L_{rodbio} - (\mu+\sigma)*B_{rodbio} - c_{Brodb}*B_{rodbio} + (1/X_{ai})*B_{rodabio} - (1/(X_b-X_{ai})*B_{rodbio})$
$d/dt(I_{rodbio}) = \sigma*B_{rodbio} - (\mu+\delta)*I_{rodbio} - c_{Irodb}*I_{rodbio} + (1/X_{ai})*I_{rodabio} - (1/(X_b-X_{ai})*I_{rodbio})$

;radbio
$d/dt(L_{radbio}) = \beta*S_{dbio}*T_{Ira}/N_0 - (\mu+\gamma)*L_{radbio} + (1/X_{ai})*L_{radabio} - (1/(X_b-X_{ai})*L_{radbio})$
$d/dt(B_{radbio}) = \gamma*L_{radbio} - (\mu+\sigma)*B_{radbio} - c_{Bradb}*B_{radbio} + (1/X_{ai})*B_{radabio} - (1/(X_b-X_{ai})*B_{radbio})$
$d/dt(I_{radbio}) = \sigma*B_{radbio} - (\mu+\delta)*I_{radbio} - c_{Iradb}*I_{radbio} + (1/X_{ai})*I_{radabio} - (1/(X_b-X_{ai})*I_{radbio})$

;rbdbio
$d/dt(L_{rbdbio}) = \beta*S_{dbio}*T_{Irb}/N_0 - (\mu+\gamma)*L_{rbdbio} + (1/X_{ai})*L_{rbdabio} - (1/(X_b-X_{ai})*L_{rbdbio})$
$d/dt(B_{rbdbio}) = \gamma*L_{rbdbio} - (\mu+\sigma)*B_{rbdbio} - c_{Brbdb}*B_{rbdbio} + (1/X_{ai})*B_{rbdabio} - (1/(X_b-X_{ai})*B_{rbdbio}) - \tau_{ab}*B_{rbdbio}$
$d/dt(I_{rbdbio}) = \sigma*B_{rbdbio} - (\mu+\delta)*I_{rbdbio} - c_{Irbdb}*I_{rbdbio} + (1/X_{ai})*I_{rbdabio} - (1/(X_b-X_{ai})*I_{rbdbio}) - \tau_{ab}*I_{rbdbio}$

$N_{dbio} = S_{dbio} + L_{rodbio} + B_{rodbio} + I_{rodbio} + L_{radbio} + B_{radbio} + I_{radbio} + L_{rbdbio} + B_{rbdbio} + I_{rbdbio}$

;Intervention 2
;MSAT using atovaquone/proguanil
; $N_{dvgpi2,}$ $N_{dvgi2,}$ $N_{dvi2,}$ $N_{doi2}$

;Box $N_{dvgpi2}$

init $S_{dvgpi2} = 0$
;init $B_{dvgpi2} = B_{dvgpi2}0$
;init $L_{dvgpi2} = L_{dvgpi2}0$
;init $I_{dvgpi2} = I_{dvgpi2}0$

$L_{dvgpi2} = L_{rodvgpi2}+L_{radvgpi2}+L_{rbdvgpi2}$
$B_{dvgpi2} = B_{rodvgpi2}+B_{radvgpi2}+B_{rbdvgpi2}$
$I_{dvgpi2} = I_{rodvgpi2}+I_{radvgpi2}+I_{rbdvgpi2}$

init $L_{rodvgpi2} = 0$
init $L_{radvgpi2} = 0$
init $L_{rbdvgpi2} = 0$
init $B_{rodvgpi2} = 0$

init $B_{radvgpi2} = 0$
init $B_{rbdvgpi2} = 0$
init $I_{rodvgpi2} = 0$
init $I_{radvgpi2} = 0$
init $I_{rbdvgpi2} = 0$

$d/dt(S_{dvgpi2}) = - \mu*S_{dvgpi2} - \beta*S_{dvgpi2}*T_{Iro}/N_0 + \delta*I_{rodvgpi2} + c_{Ldvg}*L_{rodvgpi2} + c_{Bdvg}*B_{rodvgpi2} + c_{Idvg}*I_{rodvgpi2} - \beta*S_{dvgpi2}*T_{Ira}/N_0 + \delta*I_{radvgpi2} + c_{Ldvg}*L_{radvgpi2} + c_{Bdvg}*B_{radvgpi2} + c_{Idvg}*I_{radvgpi2} - \beta*S_{dvgpi2}*T_{Irb}/N_0 + \delta*I_{rbdvgpi2} + c_{Ldvg}*L_{rbdvgpi2} + c_{Bdvg}*B_{rbdvgpi2} + c_{Idvg}*I_{rbdvgpi2} - (1/X_p)*S_{dvgpi2} + c_{Lp}*(L_{rodvgpi2} + L_{radvgpi2} + L_{rbdvgpi2}) + c_{Ip}*(I_{rodvgpi2} + I_{radvgpi2} + I_{rbdvgpi2})$

;rodvgpi2
$d/dt(L_{rodvgpi2}) = \beta*S_{dvgpi2}*T_{Iro}/N_0 - (\mu+\gamma)*L_{rodvgpi2} - (1/X_p)*L_{rodvgpi2} - c_{Lp}*L_{rodvgpi2} - c_{Ldvg}*L_{rodvgpi2}$
$d/dt(B_{rodvgpi2}) = \gamma*L_{rodvgpi2} - (\mu+\sigma)*B_{rodvgpi2} + \tau_2*B_{rodoio} + \tau_2*B_{rodaio} - (1/X_p)*B_{rodvgpi2} - c_{Bdvg}*B_{rodvgpi2} + \tau_2*B_{rodoi2} + \tau_2*B_{rodabi2} + \tau_2*B_{rodbi2}$
$d/dt(I_{rodvgpi2}) = \sigma*B_{rodvgpi2} - (\mu+\delta)*I_{rodvgpi2} + \tau_2*I_{rodoio} + \tau_2*I_{rodaio} - (1/X_p)*I_{rodvgpi2} - c_{Ip}*I_{rodvgpi2} - c_{Idvg}*I_{rodvgpi2} + \tau_2*I_{rodoi2} + \tau_2*I_{rodabi2} + \tau_2*I_{rodbi2}$

;radvgpi2
$d/dt(L_{radvgpi2}) = \beta*S_{dvgpi2}*T_{Ira}/N_0 - (\mu+\gamma)*L_{radvgpi2} - (1/X_p)*L_{radvgpi2} - c_{Lp}*L_{radvgpi2} - c_{Ldvg}*L_{radvgpi2}$
$d/dt(B_{radvgpi2}) = \gamma*L_{radvgpi2} - (\mu+\sigma)*B_{radvgpi2} + \tau_2*B_{radoio} + \tau_2*B_{radaio} - (1/X_p)*B_{radvgpi2} - c_{Bdvg}*B_{radvgpi2} + \tau_2*B_{radoi2} + \tau_2*B_{radabi2} + \tau_2*B_{radbi2}$
$d/dt(I_{radvgpi2}) = \sigma*B_{radvgpi2} - (\mu+\delta)*I_{radvgpi2} + \tau_2*I_{radoio} + \tau_2*I_{radaio} - (1/X_p)*I_{radvgpi2} - c_{Ip}*I_{radvgpi2} - c_{Idvg}*I_{radvgpi2} + \tau_2*I_{radoi2} + \tau_2*I_{radabi2} + \tau_2*I_{radbi2}$

;rbdvgpi2
$d/dt(L_{rbdvgpi2}) = \beta*S_{dvgpi2}*T_{Irb}/N_0 - (\mu+\gamma)*L_{rbdvgpi2} - (1/X_p)*L_{rbdvgpi2} - c_{Lp}*L_{rbdvgpi2} - c_{Ldvg}*L_{rbdvgpi2}$
$d/dt(B_{rbdvgpi2}) = \gamma*L_{rbdvgpi2} - (\mu+\sigma)*B_{rbdvgpi2} + \tau_2*B_{rbdoio} + \tau_2*B_{rbdaio} - (1/X_p)*B_{rbdvgpi2} - c_{Bdvg}*B_{rbdvgpi2} + \tau_2*B_{rbdoi2} + \tau_2*B_{rbdabi2} + \tau_2*B_{rbdbi2}$
$d/dt(I_{rbdvgpi2}) = \sigma*B_{rbdvgpi2} - (\mu+\delta)*I_{rbdvgpi2} + \tau_2*I_{rbdoio} + \tau_2*I_{rbdaio} - (1/X_p)*I_{rbdvgpi2} - c_{Ip}*I_{rbdvgpi2} - c_{Idvg}*I_{rbdvgpi2} + \tau_2*I_{rbdoi2} + \tau_2*I_{rbdabi2} + \tau_2*I_{rbdbi2}$

$N_{dvgpi2} = S_{dvgpi2} + L_{rodvgpi2} + B_{rodvgpi2} + I_{rodvgpi2} + L_{radvgpi2} + B_{radvgpi2} + I_{radvgpi2} + L_{rbdvgpi2} + B_{rbdvgpi2} + I_{rbdvgpi2}$

;Box $N_{dvgi2}$

init $S_{dvgi2} = 0$
;init $B_{dvgi2} = B_{dvgi2}0$
;init $L_{dvgi2} = L_{dvgi2}0$

;init $I_{dvgi2} = I_{dvgi2}0$

$L_{dvgi2} = L_{rodvgi2}+L_{radvgi2}+L_{rbdvgi2}$
$B_{dvgi2} = B_{rodvgi2}+B_{radvgi2}+B_{rbdvgi2}$
$I_{dvgi2} = I_{rodvgi2}+I_{radvgi2}+I_{rbdvgi2}$

init $L_{rodvgi2} = 0$
init $L_{radvgi2} = 0$
init $L_{rbdvgi2} = 0$
init $B_{rodvgi2} = 0$
init $B_{radvgi2} = 0$
init $B_{rbdvgi2} = 0$
init $I_{rodvgi2} = 0$
init $I_{radvgi2} = 0$
init $I_{rbdvgi2} = 0$

$d/dt(S_{dvgi2}) = - \mu*S_{dvgi2} - \beta*S_{dvgi2}*T_{Iro}/N_0 + \delta*I_{rodvgi2} - \beta*S_{dvgi2}*T_{Ira}/N_0 + \delta*I_{radvgi2} - \beta*S_{dvgi2}*T_{Irb}/N_0 + \delta*I_{rbdvgi2} + (1/X_p)*S_{dvgpi2} - (1/(X_g-X_p)*S_{dvgi2}) + c_{Ldvg}*(L_{rodvgi2} + L_{radvgi2} + L_{rbdvgi2}) + c_{Bdvg}*(B_{rodvgi2} + B_{radvgi2} + B_{rbdvgi2}) + c_{Idvg}*(I_{rodvgi2} + I_{radvgi2} + I_{rbdvgi2})$

;rodvgi2
$d/dt(L_{rodvgi2}) = \beta*S_{dvgi2}*T_{Iro}/N_0 - (\mu+\gamma)*L_{rodvgi2} + (1/X_p)*L_{rodvgpi2} - (1/(X_g-X_p)*L_{rodvgi2}) - c_{Ldvg}*L_{rodvgi2}$
$d/dt(B_{rodvgi2}) = \gamma*L_{rodvgi2} - (\mu+\sigma)*B_{rodvgi2} + (1/X_p)*B_{rodvgpi2} - (1/(X_g-X_p)*B_{rodvgi2}) - c_{Bdvg}*B_{rodvgi2}$
$d/dt(I_{rodvgi2}) = \sigma*B_{rodvgi2} - (\mu+\delta)*I_{rodvgi2} + (1/X_p)*I_{rodvgpi2} - (1/(X_g-X_p)*I_{rodvgi2}) - c_{Idvg}*I_{rodvgi2}$

;radvgi2
$d/dt(L_{radvgi2}) = \beta*S_{dvgi2}*T_{Ira}/N_0 - (\mu+\gamma)*L_{radvgi2} + (1/X_p)*L_{radvgpi2} - (1/(X_g-X_p)*L_{radvgi2}) - c_{Ldvg}*L_{radvgi2}$
$d/dt(B_{radvgi2}) = \gamma*L_{radvgi2} - (\mu+\sigma)*B_{radvgi2} + (1/X_p)*B_{radvgpi2} - (1/(X_g-X_p)*B_{radvgi2}) - c_{Bdvg}*B_{radvgi2}$
$d/dt(I_{radvgi2}) = \sigma*B_{radvgi2} - (\mu+\delta)*I_{radvgi2} + (1/X_p)*I_{radvgpi2} - (1/(X_g-X_p)*I_{radvgi2}) - c_{Idvg}*I_{radvgi2}$

;rbdvgi2
$d/dt(L_{rbdvgi2}) = \beta*S_{dvgi2}*T_{Irb}/N_0 - (\mu+\gamma)*L_{rbdvgi2} + (1/X_p)*L_{rbdvgpi2} - (1/(X_g-X_p)*L_{rbdvgi2}) - c_{Ldvg}*L_{rbdvgi2}$
$d/dt(B_{rbdvgi2}) = \gamma*L_{rbdvgi2} - (\mu+\sigma)*B_{rbdvgi2} + (1/X_p)*B_{rbdvgpi2} - (1/(X_g-X_p)*B_{rbdvgi2}) - c_{Bdvg}*B_{rbdvgi2}$
$d/dt(I_{rbdvgi2}) = \sigma*B_{rbdvgi2} - (\mu+\delta)*I_{rbdvgi2} + (1/X_p)*I_{rbdvgpi2} - (1/(X_g-X_p)*I_{rbdvgi2}) - c_{Idvg}*I_{rbdvgi2}$

$N_{dvgi2} = S_{dvgi2} + L_{rodvgi2} + B_{rodvgi2} + I_{rodvgi2} + L_{radvgi2} + B_{radvgi2} + I_{radvgi2} + L_{rbdvgi2} + B_{rbdvgi2} + I_{rbdvgi2}$

;Box $N_{dvi2}$

init $S_{dvi2} = 0$
;init $B_{dvi2} = B_{dvi2}0$
;init $L_{dvi2} = L_{dvi2}0$
;init $I_{dvi2} = I_{dvi2}0$

$L_{dvi2} = L_{rodvi2}+L_{radvi2}+L_{rbdvi2}$
$B_{dvi2} = B_{rodvi2}+B_{radvi2}+B_{rbdvi2}$
$I_{dvi2} = I_{rodvi2}+I_{radvi2}+I_{rbdvi2}$

init $L_{rodvi2} = 0$
init $L_{radvi2} = 0$
init $L_{rbdvi2} = 0$
init $B_{rodvi2} = 0$
init $B_{radvi2} = 0$
init $B_{rbdvi2} = 0$
init $I_{rodvi2} = 0$
init $I_{radvi2} = 0$
init $I_{rbdvi2} = 0$

$d/dt(S_{dvi2}) = - \mu*S_{dvi2} - \beta*S_{dvi2}*T_{Iro}/N_0 + \delta*I_{rodvi2} - \beta*S_{dvi2}*T_{Ira}/N_0 + \delta*I_{radvi2} - \beta*S_{dvi2}*T_{Irb}/N_0 + \delta*I_{rbdvi2} + (1/(X_g-X_p))*S_{dvgi2} - (1/(X_v-X_g-X_p)*S_{dvi2}) + c_{Ldv}*(L_{rodvi2} + L_{radvi2} + L_{rbdvi2}) + c_{Bdv}*(B_{rodvi2} + B_{radvi2} + B_{rbdvi2}) + c_{Idv}*(I_{rodvi2} + I_{radvi2} + I_{rbdvi2})$

;rodvi2
$d/dt(L_{rodvi2}) = \beta*S_{dvi2}*T_{Iro}/N_0 - (\mu+\gamma)*L_{rodvi2} - c_{Ldv}*L_{rodvi2} + (1/(X_g-X_p))*L_{rodvgi2} - (1/(X_v-X_g-X_p)*L_{rodvi2})$
$d/dt(B_{rodvi2}) = \gamma*L_{rodvi2} - (\mu+\sigma)*B_{rodvi2} - c_{Bdv}*B_{rodvi2} + (1/(X_g-X_p))*B_{rodvgi2} - (1/(X_v-X_g-X_p)*B_{rodvi2})$
$d/dt(I_{rodvi2}) = \sigma*B_{rodvi2} - (\mu+\delta)*I_{rodvi2} - c_{Idv}*I_{rodvi2} + (1/(X_g-X_p))*I_{rodvgi2} - (1/(X_v-X_g-X_p)*I_{rodvi2})$

;radvi2
$d/dt(L_{radvi2}) = \beta*S_{dvi2}*T_{Ira}/N_0 - (\mu+\gamma)*L_{radvi2} - c_{Ldv}*L_{radvi2} + (1/(X_g-X_p))*L_{radvgi2} - (1/(X_v-X_g-X_p)*L_{radvi2})$
$d/dt(B_{radvi2}) = \gamma*L_{radvi2} - (\mu+\sigma)*B_{radvi2} - c_{Bdv}*B_{radvi2} + (1/(X_g-X_p))*B_{radvgi2} - (1/(X_v-X_g-X_p)*B_{radvi2})$
$d/dt(I_{radvi2}) = \sigma*B_{radvi2} - (\mu+\delta)*I_{radvi2} - c_{Idv}*I_{radvi2} + (1/(X_g-X_p))*I_{radvgi2} - (1/(X_v-X_g-X_p)*I_{radvi2})$

;rbdvi2

$d/dt(L_{rbdvi2}) = \beta*S_{dvi2}*T_{Irb}/N_0 - (\mu+\gamma)*L_{rbdvi2} - c_{Ldv}*L_{rbdvi2} + (1/(X_g-X_p))*L_{rbdvgi2} - (1/(X_v-X_g-X_p)*L_{rbdvi2})$
$d/dt(B_{rbdvi2}) = \gamma*L_{rbdvi2} - (\mu+\sigma)*B_{rbdvi2} - c_{Bdv}*B_{rbdvi2} + (1/(X_g-X_p))*B_{rbdvgi2} - (1/(X_v-X_g-X_p)*B_{rbdvi2})$
$d/dt(I_{rbdvi2}) = \sigma*B_{rbdvi2} - (\mu+\delta)*I_{rbdvi2} - c_{Idv}*I_{rbdvi2} + (1/(X_g-X_p))*I_{rbdvgi2} - (1/(X_v-X_g-X_p)*I_{rbdvi2})$

$N_{dvi2} = S_{dvi2} + L_{rodvi2} + B_{rodvi2} + I_{rodvi2} + L_{radvi2} + B_{radvi2} + I_{radvi2} + L_{rbdvi2} + B_{rbdvi2} + I_{rbdvi2}$

;Box $N_{doi2}$

init $S_{doi2} = 0$
$I_{doi2}0 = 0$

$L_{doi2} = L_{rodoi2}+L_{radoi2}+L_{rbdoi2}$
$B_{doi2} = B_{rodoi2}+B_{radoi2}+B_{rbdoi2}$
$I_{doi2} = I_{rodoi2}+I_{radoi2}+I_{rbdoi2}$

init $L_{rodoi2} = 0$
init $L_{radoi2} = 0$
init $L_{rbdoi2} = 0$
init $B_{rodoi2} = 0$
init $B_{radoi2} = 0$
init $B_{rbdoi2} = 0$
init $I_{rodoi2} = 0$
init $I_{radoi2} = 0$
init $I_{rbdoi2} = 0$

$d/dt(S_{doi2}) = - \mu*S_{doi2} - \beta*S_{doi2}*T_{Iro}/N_0 + \delta*I_{rodoi2} + c_{Brodo}*B_{rodoi2} + c_{Irodo}*I_{rodoi2} - \beta*S_{doi2}*T_{Ira}/N_0 + \delta*I_{radoi2} + c_{Brado}*B_{radoi2} + c_{Irado}*I_{radoi2} - \beta*S_{doi2}*T_{Irb}/N_0 + \delta*I_{rbdoi2} + c_{Brbdo}*B_{rbdoi2} + c_{Irbdo}*I_{rbdoi2} + (1/(X_v-X_g-X_p))*S_{dvi2} + 1/(X_b-X_{ai})*S_{dbi2} + (1/X_{ao})*S_{dai2}$

;rodoi2
$d/dt(L_{rodoi2}) = \beta*S_{doi2}*T_{Iro}/N_0 - (\mu+\gamma)*L_{rodoi2} + (1/(X_v-X_g-X_p))*L_{rodvi2} + 1/(X_b-X_{ai})*L_{rodbi2} + (1/X_{ao})*L_{rodai2}$
$d/dt(B_{rodoi2}) = \gamma*L_{rodoi2} - (\mu+\sigma)*B_{rodoi2} - c_{Brodo}*B_{rodoi2} + (1/(X_v-X_g-X_p))*B_{rodvi2} - \tau_{abi2}*B_{rodoi2} + 1/(X_b-X_{ai})*B_{rodbi2} - \tau_{ai}2*B_{rodoi2} + (1/X_{ao})*B_{rodai2}-\tau_2*B_{rodoi2}$
$d/dt(I_{rodoi2}) = \sigma*B_{rodoi2} - (\mu+\delta)*I_{rodoi2} - c_{Irodo}*I_{rodoi2} + (1/(X_v-X_g-X_p))*I_{rodvi2} - \tau_{abi2}*I_{rodoi2} + 1/(X_b-X_{ai})*I_{rodbi2} - \tau_{ai2}*I_{rodoi2} + (1/X_{ao})*I_{rodai2}-\tau_2*I_{rodoi2}$

;radoi2

$d/dt(L_{radoi2}) = \beta*S_{doi2}*T_{Ira}/N_0 - (\mu+\gamma)*L_{radoi2} + (1/(X_v-X_g-X_p))*L_{radvi2} + 1/(X_b-X_{ai})*L_{radbi2} + (1/X_{ao})*L_{radai2}$
$d/dt(B_{radoi2}) = \gamma*L_{radoi2} - (\mu+\sigma)*B_{radoi2} - c_{Brado}*B_{radoi2} + (1/(X_v-X_g-X_p))*B_{radvi2} - \tau_{abi2}*B_{radoi2} + 1/(X_b-X_{ai})*B_{radbi2} - \tau_{ai2}*B_{radoi2} + (1/X_{ao})*B_{radai2}-\tau_2*B_{radoi2}$
$d/dt(I_{radoi2}) = \sigma*B_{radoi2} - (\mu+\delta)*I_{radoi2} - c_{Irado}*I_{radoi2} + (1/(X_v-X_g-X_p))*I_{radvi2} - \tau_{abi2}*I_{radoi2} + 1/(X_b-X_{ai})*I_{radbi2} - \tau_{ai2}*I_{radoi2} + (1/X_{ao})*I_{radai2}-\tau_2*I_{radoi2}$

;rbdoi2
$d/dt(L_{rbdoi2}) = \beta*S_{doi2}*T_{Irb}/N_0 - (\mu+\gamma)*L_{rbdoi2} + (1/(X_v-X_g-X_p))*L_{rbdvi2} + 1/(X_b-X_{ai})*L_{rbdbi2} + (1/X_{ao})*L_{rbdai2}$
$d/dt(B_{rbdoi2}) = \gamma*L_{rbdoi2} - (\mu+\sigma)*B_{rbdoi2} - c_{Brbdo}*B_{rbdoi2} + (1/(X_v-X_g-X_p))*B_{rbdvi2} - \tau_{abi2}*B_{rbdoi2} + 1/(X_b-X_{ai})*B_{rbdbi2} - \tau_{ai2}*B_{rbdoi2} + (1/X_{ao})*B_{rbdai2}-\tau_2*B_{rbdoi2}$
$d/dt(I_{rbdoi2}) = \sigma*B_{rbdoi2} - (\mu+\delta)*I_{rbdoi2} - c_{Irbdo}*I_{rbdoi2} + (1/(X_v-X_g-X_p))*I_{rbdvi2} - \tau_{abi2}*I_{rbdoi2} + 1/(X_b-X_{ai})*I_{rbdbi2} - \tau_{ai2}*I_{rbdoi2} + (1/X_{ao})*I_{rbdai2}-\tau_2*I_{rbdoi2}$

$N_{doi2} = S_{doi2} + L_{rodoi2} + B_{rodoi2} + I_{rodoi2} + L_{radoi2} + B_{radoi2} + I_{radoi2} + L_{rbdoi2} + B_{rbdoi2} + I_{rbdoi2}$

;Treatment with artesunate in intervention 2
; $N_{dai2}$

;Box $N_{dai2}$

init $S_{dai2} = 0$
;init $B_{dai2} = B_{dai2}0$
;init $L_{dai2} = L_{dai2}0$
;init $I_{dai2} = I_{dai2}0$

$L_{dai2} = L_{rodai2}+L_{radai2}+L_{rbdai2}$
$B_{dai2} = B_{rodai2}+B_{radai2}+B_{rbdai2}$
$I_{dai2} = I_{rodai2}+I_{radai2}+I_{rbdai2}$

init $L_{rodai2} = 0$
init $L_{radai2} = 0$
init $L_{rbdai2} = 0$
init $B_{rodai2} = 0$
init $B_{radai2} = 0$
init $B_{rbdai2} = 0$
init $I_{rodai2} = 0$
init $I_{radai2} = 0$
init $I_{rbdai2} = 0$

$d/dt(S_{dai2}) = - \mu*S_{dai2} - \beta*S_{dai2}*T_{Iro}/N_0 + \delta*I_{rodai2} + c_{Broda}*B_{rodai2} + c_{Iroda}*I_{rodai2} - \beta*S_{dai2}*T_{Ira}/N_0 + \delta*I_{radai2} + c_{Brada}*B_{radai2} + c_{Irada}*I_{radai2} - \beta*S_{dai2}*T_{Irb}/N_0 + \delta*I_{rbdai2} + c_{Brbda}*B_{rbdai2} + c_{Irbda}*I_{rbdai2} - (1/X_{ao})*S_{dai2}$

;rodai2
$d/dt(L_{rodai2}) = \beta*S_{dai2}*T_{Iro}/N_0 - (\mu+\gamma)*L_{rodai2} - (1/X_{ao})*L_{rodai2}$
$d/dt(B_{rodai2}) = \gamma*L_{rodai2} - (\mu+\sigma)*B_{rodai2} - c_{Broda}*B_{rodai2} + \tau_{ai2}*B_{rodoi2} - (1/X_{ao})*B_{rodai2}$
$d/dt(I_{rodai2}) = \sigma*B_{rodai2} - (\mu+\delta)*I_{rodai2} - c_{Iroda}*I_{rodai2} + \tau_{ai2}*I_{rodoi2} - (1/X_{ao})*I_{rodai2}$

;radai2
$d/dt(L_{radai2}) = \beta*S_{dai2}*T_{Ira}/N_0 - (\mu+\gamma)*L_{radai2} - (1/X_{ao})*L_{radai2}$
$d/dt(B_{radai2}) = \gamma*L_{radai2} - (\mu+\sigma)*B_{radai2} - c_{Brada}*B_{radai2} + \tau_{ai2}*B_{radoi2} - (1/X_{ao})*B_{radai2}$
$d/dt(I_{radai2}) = \sigma*B_{radai2} - (\mu+\delta)*I_{radai2} - c_{Irada}*I_{radai2} + \tau_{ai2}*I_{radoi2} - (1/X_{ao})*I_{radai2}$

;rbdai2
$d/dt(L_{rbdai2}) = \beta*S_{dai2}*T_{Irb}/N_0 - (\mu+\gamma)*L_{rbdai2} - (1/X_{ao})*L_{rbdai2}$
$d/dt(B_{rbdai2}) = \gamma*L_{rbdai2} - (\mu+\sigma)*B_{rbdai2} - c_{Brbda}*B_{rbdai2} + \tau_{ai2}*B_{rbdoi2} - (1/X_{ao})*B_{rbdai2}$
$d/dt(I_{rbdai2}) = \sigma*B_{rbdai2} - (\mu+\delta)*I_{rbdai2} - c_{Irbda}*I_{rbdai2} + \tau_{ai2}*I_{rbdoi2} - (1/X_{ao})*I_{rbdai2}$

$N_{dai2} = S_{dai2} + L_{rodai2} + B_{rodai2} + I_{rodai2} + L_{radai2} + B_{radai2} + I_{radai2} + L_{rbdai2} + B_{rbdai2} + I_{rbdai2}$

;Treatment with ACT (artesunate + piperaquine) during intervention 2
; $N_{dabi2,}$ $N_{dbi2}$

;Box $N_{dabi2}$

init $S_{dabi2} = 0$
;init $B_{dabi2} = B_{dabi2}0$
;init $L_{dabi2} = L_{dabi2}0$
;init $I_{dabi2} = I_{dabi2}0$

$L_{dabi2} = L_{rodabi2}+Lra_{dabi2}+L_{rbdabi2}$
$B_{dabi2} = B_{rodabi2}+Bra_{dabi2}+B_{rbdabi2}$
$I_{dabi2} = I_{rodabi2}+I_{radabi2}+I_{rbdabi2}$

init $L_{rodabi2} = 0$
init $Lra_{dabi2} = 0$
init $L_{rbdabi2} = 0$
init $B_{rodabi2} = 0$
init $Bra_{dabi2} = 0$
init $B_{rbdabi2} = 0$
init $I_{rodabi2} = 0$
init $I_{radabi2} = 0$
init $I_{rbdabi2} = 0$

$d/dt(S_{dabi2}) = - \mu*S_{dabi2} - \beta*S_{dabi2}*T_{Iro}/N_0 + \delta*I_{rodabi2} + c_{Brodab}*B_{rodabi2} + c_{Irodab}*I_{rodabi2} - \beta*S_{dabi2}*T_{Ira}/N_0 + \delta*I_{radabi2} + c_{Bradab}*B_{radabi2} + c_{Iradab}*I_{radabi2} - \beta*S_{dabi2}*T_{Irb}/N_0 + \delta*I_{rbdabi2} + c_{Brbdab}*B_{rbdabi2} + c_{Irbdab}*I_{rbdabi2} - (1/X_{ai}*S_{dabi2})$

;rodabi2
$d/dt(L_{rodabi2}) = \beta*S_{dabi2}*T_{Iro}/N_0 - (\mu+\gamma)*L_{rodabi2} - (1/X_{ai})*L_{rodabi2}$
$d/dt(B_{rodabi2}) = \gamma*L_{rodabi2} - (\mu+\sigma)*B_{rodabi2} - c_{Brodab}*B_{rodabi2} - (1/X_{ai})*B_{rodabi2} + \tau_{abi2}*B_{rodoi2} - \tau_2*B_{rodabi2}$
$d/dt(I_{rodabi2}) = \sigma*B_{rodabi2} - (\mu+\delta)*I_{rodabi2} - c_{Irodab}*I_{rodabi2} - (1/X_{ai})*I_{rodabi2} + \tau_{abi2}*I_{rodoi2} - \tau_2*I_{rodabi2}$

;radabi2
$d/dt(L_{radabi2}) = \beta*S_{dabi2}*T_{Ira}/N_0 - (\mu+\gamma)*L_{radabi2} - (1/X_{ai})*L_{radabi2}$
$d/dt(B_{radabi2}) = \gamma*L_{radabi2} - (\mu+\sigma)*B_{radabi2} - c_{Bradab}*B_{radabi2} - (1/X_{ai})*B_{radabi2} + \tau_{abi2}*B_{radoi2} - \tau_2*B_{radabi2}$
$d/dt(I_{radabi2}) = \sigma*B_{radabi2} - (\mu+\delta)*I_{radabi2} - c_{Iradab}*I_{radabi2} - (1/X_{ai})*I_{radabi2} + \tau_{abi2}*I_{radoi2} - \tau_2*I_{radabi2}$

;rbdabi2
$d/dt(L_{rbdabi2}) = \beta*S_{dabi2}*T_{Irb}/N_0 - (\mu+\gamma)*L_{rbdabi2} - (1/X_{ai})*L_{rbdabi2}$
$d/dt(B_{rbdabi2}) = \gamma*L_{rbdabi2} - (\mu+\sigma)*B_{rbdabi2} - c_{Brbdab}*B_{rbdabi2} + \tau_{ab}*B_{rbdbi2} - (1/X_{ai})*B_{rbdabi2} + \tau_{abi2}*B_{rbdoi2} - \tau_2*B_{rbdabi2}$
$d/dt(I_{rbdabi2}) = \sigma*B_{rbdabi2} - (\mu+\delta)*I_{rbdabi2} - c_{Irbdab}*I_{rbdabi2} + \tau_{ab}*I_{rbdbi2} - (1/X_{ai})*I_{rbdabi2} + \tau_{abi2}*I_{rbdoi2} - \tau_2*I_{rbdabi2}$

$N_{dabi2} = S_{dabi2} + L_{rodabi2} + B_{rodabi2} + I_{rodabi2} + Lra_{dabi2} + B_{radabi2} + I_{radabi2} + L_{rbdabi2} + B_{rbdabi2} + I_{rbdabi2}$

;Box $N_{dbi2}$

init $S_{dbi2} = 0$
;init $B_{dbi2} = B_{dbi2}0$
;init $L_{dbi2} = L_{dbi2}0$
;init $I_{dbi2} = I_{dbi2}0$

$L_{dbi2} = L_{rodbi2}+L_{radbi2}+L_{rbdbi2}$
$B_{dbi2} = B_{rodbi2}+B_{radbi2}+B_{rbdbi2}$
$I_{dbi2} = I_{rodbi2}+I_{radbi2}+I_{rbdbi2}$

init $L_{rodbi2} = 0$
init $L_{radbi2} = 0$
init $L_{rbdbi2} = 0$
init $B_{rodbi2} = 0$
init $B_{radbi2} = 0$

init $B_{rbdbi2} = 0$
init $I_{rodbi2} = 0$
init $I_{radbi2} = 0$
init $I_{rbdbi2} = 0$

d/dt($S_{dbi2}$) = - $\mu$*$S_{dbi2}$ - $\beta$*$S_{dbi2}$*$T_{Iro}$/$N_0$ + $\delta$*$I_{rodbi2}$ + $c_{Brodb}$*$B_{rodbi2}$ + $c_{Irodb}$*$I_{rodbi2}$ - $\beta$*$S_{dbi2}$*$T_{Ira}$/$N_0$ + $\delta$*$I_{radbi2}$ + $c_{Bradb}$*$B_{radbi2}$ + $c_{Iradb}$*$I_{radbi2}$ - $\beta$*$S_{dbi2}$*$T_{Irb}$/$N_0$ + $\delta$*$I_{rbdbi2}$ + $c_{Brbdb}$*$B_{rbdbi2}$ + $c_{Irbdb}$*$I_{rbdbi2}$ + (1/$X_{ai}$*$S_{dabi2}$) - 1/($X_b$-$X_{ai}$)*$S_{dbi2}$

;rodbi2
d/dt($L_{rodbi2}$) = $\beta$*$S_{dbi2}$*$T_{Iro}$/$N_0$ - ($\mu$+$\gamma$)*$L_{rodbi2}$ + (1/$X_{ai}$)*$L_{rodabi2}$ - 1/($X_b$-$X_{ai}$)*$L_{rodbi2}$
d/dt($B_{rodbi2}$) = $\gamma$*$L_{rodbi2}$ - ($\mu$+$\sigma$)*$B_{rodbi2}$ - $c_{Brodb}$*$B_{rodbi2}$ + (1/$X_{ai}$)*$B_{rodabi2}$ - 1/($X_b$-$X_{ai}$)*$B_{rodbi2}$ - $\tau_2$*$B_{rodbi2}$
d/dt($I_{rodbi2}$) = $\sigma$*$B_{rodbi2}$ - ($\mu$+$\delta$)*$I_{rodbi2}$ - $c_{Irodb}$*$I_{rodbi2}$ + (1/$X_{ai}$)*$I_{rodabi2}$ - 1/($X_b$-$X_{ai}$)*$I_{rodbi2}$ - $\tau_2$*$I_{rodbi2}$

;radbi2
d/dt($L_{radbi2}$) = $\beta$*$S_{dbi2}$*$T_{Ira}$/$N_0$ - ($\mu$+$\gamma$)*$L_{radbi2}$ + (1/$X_{ai}$)*$L_{radabi2}$ - 1/($X_b$-$X_{ai}$)*$L_{radbi2}$
d/dt($B_{radbi2}$) = $\gamma$*$L_{radbi2}$ - ($\mu$+$\sigma$)*$B_{radbi2}$ - $c_{Bradb}$*$B_{radbi2}$ + (1/$X_{ai}$)*$B_{radabi2}$ - 1/($X_b$-$X_{ai}$)*$B_{radbi2}$ - $\tau_2$*$B_{radbi2}$
d/dt($I_{radbi2}$) = $\sigma$*$B_{radbi2}$ - ($\mu$+$\delta$)*$I_{radbi2}$ - $c_{Iradb}$*$I_{radbi2}$ + (1/$X_{ai}$)*$I_{radabi2}$ - 1/($X_b$-$X_{ai}$)*$I_{radbi2}$ - $\tau_2$*$I_{radbi2}$

;rbdbi2
d/dt($L_{rbdbi2}$) = $\beta$*$S_{dbi2}$*$T_{Irb}$/$N_0$ - ($\mu$+$\gamma$)*$L_{rbdbi2}$ + (1/$X_{ai}$)*$L_{rbdabi2}$ - 1/($X_b$-$X_{ai}$)*$L_{rbdbi2}$
d/dt($B_{rbdbi2}$) = $\gamma$*$L_{rbdbi2}$ - ($\mu$+$\sigma$)*$B_{rbdbi2}$ - $c_{Brbdb}$*$B_{rbdbi2}$ - $\tau_{ab}$*$B_{rbdbi2}$ + (1/$X_{ai}$)*$B_{rbdabi2}$ - 1/($X_b$-$X_{ai}$)*$B_{rbdbi2}$ - $\tau_2$*$B_{rbdbi2}$
d/dt($I_{rbdbi2}$) = $\sigma$*$B_{rbdbi2}$ - ($\mu$+$\delta$)*$I_{rbdbi2}$ - $c_{Irbdb}$*$I_{rbdbi2}$ - $\tau_{ab}$*$I_{rbdbi2}$ + (1/$X_{ai}$)*$I_{rbdabi2}$ - 1/($X_b$-$X_{ai}$)*$I_{rbdbi2}$ - $\tau_2$*$I_{rbdbi2}$

$N_{dbi2} = S_{dbi2} + L_{rodbi2} + B_{rodbi2} + I_{rodbi2} + L_{radbi2} + B_{radbi2} + I_{radbi2} + L_{rbdbi2} + B_{rbdbi2} + I_{rbdbi2}$

;CHECKS AND TOTALS

check = $N_0$ - ($N_{doio}$ + $N_{daio}$ + $N_{dabi1}$ + $N_{dbi1}$ + $N_{doi1}$ + $N_{dabio}$ + $N_{dbio}$ + $Nd_{ai1a}$ + $N_{dabi1a}$ + $N_{dbi1a}$ + $N_{dvgpi2}$ + $N_{dvgi2}$ + $N_{dvi2}$ + $N_{doi2}$ + $N_{dai2}$ + $N_{dabi2}$ + $N_{dbi2}$)

$T_N$ = $N_{doio}$ + $N_{daio}$ +$N_{dabi1}$ + $N_{dbi1}$ + $N_{doi1}$ + $N_{dabio}$ + $N_{dbio}$ + $Nd_{ai1a}$ + $N_{dabi1a}$ + $N_{dbi1a}$ + $N_{dvgpi2}$ + $N_{dvgi2}$ + $N_{dvi2}$ + $N_{doi2}$ + $N_{dai2}$ + $N_{dabi2}$ + $N_{dbi2}$

$N_{ra} = (I_{radoio} + I_{radaio} + I_{radabi1} + I_{radbi1} + I_{radoi1} + I_{radabio} + I_{radbio} + I_{radai1a} + I_{radabi1a} + I_{radbi1a}) + (B_{radoio} + B_{radaio} + B_{radabi1} + B_{radbi1} + B_{radoi1} + B_{radabio} + B_{radbio} + B_{radai1a} + B_{radabi1a} + B_{radbi1a}) + (I_{radvgpi2} + I_{radvgi2} + I_{radvi2} + I_{radoi2} + I_{radai2} + I_{radabi2} + I_{radbi2}) + (B_{radvgpi2} + B_{radvgi2} + B_{radvi2} + B_{radoi2} + B_{radai2} + B_{radabi2} + B_{radbi2})$

$N_{rb} = (I_{rbdoio} + I_{rbdaio} + I_{rbdabi1} + I_{rbdbi1} + I_{rbdoi1} + I_{rbdabio} + I_{rbdbio} + I_{rbdai1a} + I_{rbdabi1a} + I_{rbdbi1a}) + (B_{rbdai1a} + B_{rbdoio} + B_{rbdaio} + B_{rbdabi1} + B_{rbdbi1} + B_{rbdoi1} + B_{rbdabio} + B_{rbdbio} + B_{rbdai1a} + B_{rbdabi1a} + B_{rbdbi1a}) + (I_{rbdvgpi2} + I_{rbdvgi2} + I_{rbdvi2} + I_{rbdoi2} + I_{rbdai2} + I_{rbdabi2} + I_{rbdbi2}) + (B_{rbdai2} + B_{rbdvgpi2} + B_{rbdvgi2} + B_{rbdvi2} + B_{rbdoi2} + B_{rbdai2} + B_{rbdabi2} + B_{rbdbi2})$

$T_{IB} = (I_{doio} + I_{daio} + I_{dabi1} + I_{dbi1} + I_{doi1} + I_{dabio} + I_{dbio} + I_{dai1a} + I_{dabi1a} + I_{dbi1a}) + (B_{doio} + B_{daio} + B_{dabi1} + B_{dbi1} + B_{doi1} + B_{dabio} + B_{dbio} + B_{dai1a} + B_{dabi1a} + B_{dbi1a}) + (I_{dvgpi2} + I_{dvgi2} + I_{dvi2} + I_{doi2} + I_{dai2} + I_{dabi2} + I_{dbi2}) + (B_{dvgpi2} + B_{dvgi2} + B_{dvgi2} + B_{doi2} + B_{dai2} + B_{dabi2} + B_{dbi2})$

$\beta = amp*((1-bn)*\beta_n)*\cos(2*3.14159*(time-\pi)) + ((1-bn)*\beta_n)$
$\pi = 0.5$ ; peak time for malaria transmission with 0=January
amp = 0.67 ; amplitude of seasonal variation with value 0 to 1

$q=(\mu+\gamma)*(\mu+\sigma)*(\mu+\delta)/(\sigma*\gamma)$

$cost_a = 0$; cost of resistance to artesunate
$cost_b = 0$; cost of resistance to piperaquine

$T_{Iro} = I_{rodoio} + I_{rodaio} + I_{rodabi1} + I_{rodbi1} + I_{rodoi1} + I_{rodabio} + I_{rodbio} + I_{rodai1a} + I_{radabi1a} + I_{radbi1a} + I_{rodvgpi2} + I_{rodvgi2} + I_{rodvi2} + I_{rodoi2} + I_{rodai2} + I_{radabi2} + I_{radbi2}$
$T_{Ira} = (1-cost_a)*(I_{radoio} + I_{radaio} + I_{radabi1} + I_{radbi1} + I_{radoi1} + I_{radabio} + I_{radbio} + I_{radai1a} + I_{radabi1a} + I_{radbi1a} + I_{radvgpi2} + I_{radvgi2} + I_{radvi2} + I_{radoi2} + I_{radai2} + I_{radabi2} + I_{radbi2})$
$T_{Irb} = (1-cost_b)*(I_{rbdoio} + I_{rbdaio} + I_{rbdabi1} + I_{rbdbi1} + I_{rbdoi1} + I_{rbdabio} + I_{rbdbio} + I_{rbdai1a} + I_{rbdabi1a} + I_{rbdbi1a} + I_{rbdvgpi2} + I_{rbdvgi2} + I_{rbdvi2} + I_{rbdoi2} + I_{rbdai2} + I_{rbdabi2} + I_{rbdbi2})$
$T_I=T_{Iro}+T_{Ira}+T_{Irb}$

$T_{i1cum}' = \tau 1*(N_{doio} + N_{daio})$
$T_{i1cumcov} = (100*T_{i1cum})/T_N$
init $T_{i1cum} = 0$

$perc_{ra} = 100*N_{ra}/T_{IB}$ ; percent of blood stage infections resistant to artesunate
$perc_{rb} = 100*N_{rb}/T_{IB}$ ; percent of blood stage infections resistant to piperaquine
$perc_{IB} = 100*T_{IB}/T_N$ ; percent of infections that are blood stage

$init(IT_{roda})=0$
$IT_{roda}'=B_{rodaio} + B_{rodai1a} + L_{rodaio} + L_{rodai1a} + I_{rodaio} + I_{rodai1a} + B_{rodabio} + B_{rodabi1} + B_{rodabi1a} + L_{rodabio} + L_{rodabi1} + L_{rodabi1a} + I_{rodabio} + I_{rodabi1} + I_{rodabi1a} + B_{rodabi2} + L_{rodabi2} + I_{rodabi2} + B_{rodai2} + L_{rodai2} + I_{rodai2}$

$init(IT_{rodb})=0$

$IT_{rodb}' = B_{rodbio} + B_{rodbi1} + B_{rodbi1a} + L_{rodbio} + L_{rodbi1} + L_{rodbi1a} + I_{rodbio} + I_{rodbi1} + I_{rodbi1a} + B_{rodabio} + B_{rodabi1} +$
$B_{rodabi1a} + L_{rodabio} + L_{rodabi1} + L_{rodabi1a} + I_{rodabio} + I_{rodabi1} + I_{rodabi1a} + I_{rodabi1a} + B_{rodabi2} + L_{rodabi2} + I_{rodabi2} +$
$B_{rodbi2} + L_{rodbi2} + I_{rodbi2}$

$init(IT_{rada})=0$
$IT_{rada}' = B_{radaio} + B_{radai1a} + L_{radaio} + L_{radai1a} + I_{radaio} + I_{radai1a} + B_{radabio} + B_{radabi1} + B_{radabi1a} + L_{radabio} +$
$L_{radabi1} + L_{radabi1a} + I_{radabio} + I_{radabi1} + I_{radabi1a} + B_{radabi2} + L_{radabi2} + I_{radabi2} + B_{radai2} + L_{radai2} + I_{radai2}$

$init(IT_{radb})=0$
$IT_{radb}' = B_{radbio} + B_{radbi1} + B_{radbi1a} + L_{radbio} + L_{radbi1} + L_{radbi1a} + I_{radbio} + I_{radbi1} + I_{radbi1a} + B_{radabio} + B_{radabi1} +$
$B_{radabi1a} + L_{radabio} + L_{radabi1} + L_{radabi1a} + I_{radabio} + I_{radabi1} + I_{radabi1a} + B_{radabi2} + L_{radabi2} + I_{radabi2} + B_{radbi2} +$
$L_{radbi2} + I_{radbi2}$

$init(IT_{rbda})=0$
$IT_{rbda}' = B_{rbdaio} + B_{rbdai1a} + L_{rbdaio} + L_{rbdai1a} + I_{rbdaio} + I_{rbdai1a} + B_{rbdabio} + B_{rbdabi1} + B_{rbdabi1a} + L_{rbdabio} +$
$L_{rbdabi1} + L_{rbdabi1a} + I_{rbdabio} + I_{rbdabi1} + I_{rbdabi1a} + B_{rbdabi2} + L_{rbdabi2} + I_{rbdabi2} + B_{rbdai2} + L_{rbdai2} + I_{rbdai2}$

$init(IT_{rbdb})=0$
$IT_{rbdb}' = B_{rbdbio} + B_{rbdbi1} + B_{rbdbi1a} + L_{rbdbio} + L_{rbdbi1} + L_{rbdbi1a} + I_{rbdbio} + I_{rbdbi1} + I_{rbdbi1a} + B_{rbdabio} + B_{rbdabi1} +$
$B_{rbdabi1a} + L_{rbdabio} + L_{rbdabi1} + L_{rbdabi1a} + I_{rbdabio} + I_{rbdabi1} + I_{rbdabi1a} + B_{rbdabi2} + L_{rbdabi2} + I_{rbdabi2} + B_{rbdbi2} +$
$L_{rbdbi2} + I_{rbdbi2}$
